# A Room‐Temperature Terahertz Photodetector Imaging with High Stability and Polarization‐Sensitive Based on Perovskite/Metasurface

**DOI:** 10.1002/advs.202407634

**Published:** 2024-12-17

**Authors:** Yifan Li, Yiming Jia, He Yang, Yinghui Wu, Yajun Cao, Xuyang Zhang, Cunguang Lou, Xiuling Liu, Long‐Biao Huang, Jianquan Yao

**Affiliations:** ^1^ College of Electronic Information Engineering & Hebei Key Laboratory of Digital Medical Engineering Hebei University Baoding 071000 China; ^2^ School of Precision Instruments and Opto‐Electronics Engineering Tianjin University Tianjin 300072 China; ^3^ Key Laboratory of Optoelectronic Devices and Systems of Ministry of Education and Guangdong Province College of Physics and Optoelectronic Engineering Shenzhen University Shenzhen 518060 China; ^4^ National Key Laboratory of Green and Long‐Life Road Engineering in Extreme Environment Shenzhen University Shenzhen 518060 China

**Keywords:** metasurface, perovskite, stability, thermoelectric effect, THz polarization detection

## Abstract

Terahertz (THz) polarization detection facilitates the capture of multidimensional data, including intensity, phase, and polarization state, with broad applicability in high‐resolution imaging, communication, and remote sensing. However, conventional semiconductor materials are limited by energy band limitations, rendering them unsuitable for THz detection. Overcoming this challenge, the realization of high‐stability, room‐temperature polarization‐sensitive THz photodetectors (PDs) leveraging the thermoelectric effect of Cs_0.05_(FA_0.85_MA_0.15_)_0.95_Pb(I_0.85_Br_0.15_)_3_ (CsFAMA)/metasurfaces is presented. Two different structures of (T‐shaped and I‐shaped) THz PDs are constructed. The incorporation of perovskite/metasurfaces forms enhanced local field thermoelectric effect and polarization response. Owning to THz surface plasmon polariton (SPP) resonance effect and more boundary effect, the I‐shaped PDs exhibit superior performance, achieving a response of up to 94 V/W, with a response time of 138 µs, a low noise‐equivalent power of 5.03 pW/Hz^1/2^ and an anisotropy ratio of 1.38 under 0.1THz laser irradiation. Furthermore, the PD's stability is verified with the anisotropy ratio decreased by only 2% and polarization imaging results after 240 days of storage in air condition. This research introduces a method for achieving high‐performance, stable THz polarization detection technology, with significant potential for advancements in materials science, communication technology, and medical imaging.

## Graphical abstract

CsFAMA PDs achieve polarization‐sensitive detection capabilities within the 0.1THz. Due to the surface plasmon polariton (SPP) effect and polarization anisotropy regulator of metasurfaces, the devices exhibit a high photoresponse of 94 V/W and an anisotropy ratio of 1.38. Even after exposure to air conditioning for 240 days, performance degradation is limited to only 2%, and high‐resolution imaging results are obtained. This research offers an approach to achieving high‐performance and stable THz polarization detection technology.

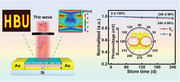

## 1 Introduction

Terahertz (THz) polarization‐sensitive detection offers a comprehensive of multidimensional information, encompassing intensity, polarization type, and orientation angle, thereby significantly enhancing detection sensitivity and dimensionality.^[^ ^1^, ^2^ ^]^ This capability exhibits considerable promise for applications in communications, radar, high‐resolution imaging, and beyond.^[^ ^3^, ^4^, ^5^, ^6^ ^]^ Currently, THz detection predominantly utilizes the principles of photoelectric, nonlinear optical, and photothermoelectric (PTE) effects.^[^ ^7^, ^8^, ^9^ ^]^ In particular, THz PTE detectors have attracted considerable attention owing to their ability to operate effectively at room‐temperature, wavelength non‐selectivity, and rapid response.^[^ ^10^, ^11^, ^12^ ^]^ There is a compelling need for high‐stability, polarization‐sensitive THz PDs that can acquire multidimensional information at room‐temperature.^[^ ^13^, ^14^, ^15^, ^16^ ^]^ Previous studies have demonstrated that polarization information can be extracted through traditional polarization photodetection systems utilizing optical media featuring plasmonic microcavity structures or quantum well configurations.^[^ ^17^, ^18^ ^]^ Nevertheless, these systems are hampered by large volume, structural complexity, and limited applicability across diverse fields.^[^ ^19^ ^]^

Leveraging micro‐nano technology, metasurfaces provide versatile and effective management over THz waves, including polarization, amplitude, and phase modulation. This capability amplifies polarization responsiveness while reducing the footprint of optical systems effectively.^[^ ^20^, ^21^, ^22^ ^]^ Researchers have investigated diverse strategies for fabricating THz polarization modulable devices utilizing a 3D folding metasurface, phase control metasurface, and geometry symmetry metasurface.^[^ ^23^, ^24^, ^25^ ^]^ Moreover, THz surface plasmon polariton (SPP) resonance within metasurfaces induces a local thermoelectric field, thereby enhancing PTE detection efficiency and THz response. However, achieving high sensitivity and rapid response for polarization detection remains a great challenge.

In recent years, the anisotropic semiconductor perovskite material CH_3_NH_3_PbI_3_ (MAPbI_3_) has emerged significant attention as a polarization detector due to its notable attributes such as high absorption coefficient, carrier mobility, and intrinsic anisotropy.^[^ ^26^, ^27^ ^]^ Motivated by its exceptional photoelectric and anisotropy properties, several scientific groups have successfully demonstrated linear and circular polarization through the fabrication of 2D/3D MAPbI_3_ heterojunctions, ion doping MAPbI_3,_ and all‐dielectric MAPbI_3_ metasurfaces.^[^ ^28^, ^29^, ^30^ ^]^ Despite significant advancements in perovskite polarization detection, these materials are currently constrained to the visible to infrared range. Their application in the THz band is hindered by bandwidth constraints and insufficient stability.^[^ ^31^, ^32^ ^]^ Previous studies have reported advanced 2D material preparation methods ^[^ ^33^, ^34^ ^]^ and that substituting MA^+^ cation with the FA^+^ and incorporating mixed‐halide compositions with I and Br can enhance the thermal and moisture stability of the device, particularly under high‐humidity environments.^[^ ^35^, ^36^ ^]^

Herein, we investigated highly stable and polarization‐sensitive THz PDs designed with a composite structure composed of (CsFAMA) / Poly (3,4‐ethylenedioxythiophene) poly(styrenesulfonate) (PEDOT: PSS)/metasurfaces, exploiting the PTE effect. Two distinct THz polarization‐sensitive PD designs are developed with T‐shaped and I‐shaped configurations. The design of metasurface/CsFAMA not only regulates the THz polarization response characteristics but also facilitates the formation of SPP, thereby enhancing THz absorption.^[^ ^37^, ^38^, ^39^, ^40^ ^]^ The CsFAMA/PEDOT: PSS heterojunction promotes the separation of hot electrons, improving the photoelectric conversion efficiency of the device.^[^ ^41^, ^42^, ^43^, ^44^ ^]^ Compared to MAPbI_3_ and other perovskites, CsFAMA exhibits higher carrier mobility, greater stability, and greater intrinsic anisotropy. Additionally, PEDOT: PSS offers excellent film‐forming properties and adhesion, enabling the formation of a uniform layer at material interfaces. This improves interface contact, prevents direct contact between the perovskite and gold, and enhances the overall stability of the device.

Moreover, by harnessing the thermoelectric with anisotropy properties, the device transcends band limitations, facilitating polarization detection and demonstrating outstanding performance across the THz spectrum. Under 0.1THz laser irradiation, the I‐shaped PDs exhibit a higher response due to the surface plasmon polariton (SPP) effect of metasurfaces, achieving a responsivity of up to 94 V/W, a noise equivalent power of 5.03 pW/Hz^1/2^ and an anisotropy ratio of 1.38. Additionally, it boasts a rapid response time of 138 µs at room‐temperature. Stability tests reveal a minimal 2% decrease in the anisotropy ratio after exposure to air conditioning for 240 days. Finally, the polarization imaging demonstration under 0.1 THz polarized illumination verifies the prospect of the device in polarization imaging. This research opens up an avenue to develop THz PDs for high‐performance stable polarization‐sensitive photodetection and next‐generation polarized imaging.

## 2 Results and Discussion

**Figure 1:**
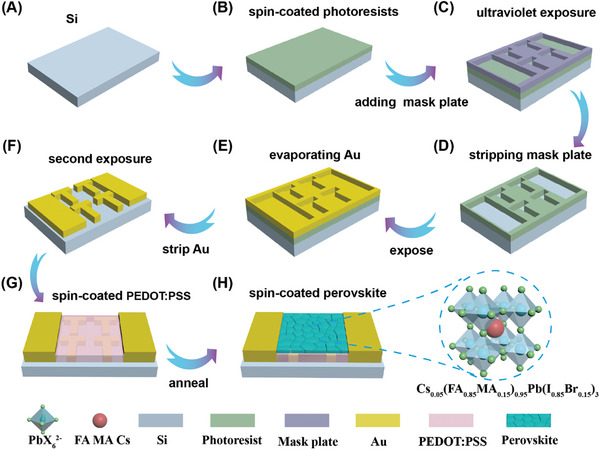
Schematic diagram of a process for preparing CsFAMA/PEDOT:PSS/ metasurfaces PDs.

The step‐by‐step preparation process of CsFAMA PDs is depicted in **Figure** **1**. Initially, the Si substrate was precisely cut to a specific size and subjected to a thorough 15 min ultrasonic cleaning in ethanol, acetone, and isopropanol. Subsequently, UV‐ozone treatment was applied for 10 min. Following this, a layer of photoresist was spin‐coated onto the Si substrate and allowed to dry. A pre‐designed mask was then applied to the photoresist‐coated substrate, which was exposed to ultraviolet light for a specific duration, resulting in the desired PDs shape within the photoresist layer. The development process took 15 min, followed by metal deposition. After metal deposition, the photoresist layer was removed, leaving behind a specific pattern of the metal layer. A second exposure was conducted, and PEDOT: PSS solution was spin‐coated at a speed of 2000 rpm. The substrate was then heated at 150°C until film formation. Finally, CsFAMA was spin‐coated onto the PEDOT: PSS film. Further details on the synthesis of the CsFAMA, device simulation characterization, and testing can be found in the Supporting Information.

**Figure 2:**
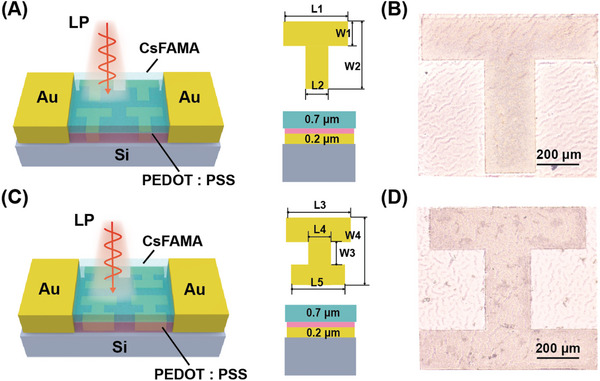
A,C) Schematic diagram of T‐shaped and I‐shaped PDs structures and dimension, respectively; B,D) Electron microgram of T‐shaped and I‐shaped metasurface, respectively.

To optimize light absorption, the structure of “Active Layer‐ PEDOT: PSS‐Metal Layer‐Substrate” for the PDs was designed. The partial periodic dimensions of the perovskite metasurface array are shown in **Figure** **2**A,C. The active layer, consisting of CsFAMA perovskite with a thickness of 0.7 µm, offers superior stability, higher charge carrier mobility, and greater intrinsic anisotropy compared to other perovskite materials. The metal layer utilizes lossy gold material with a thickness of 0.2 µm. The substrate is composed of low‐transmittance Si material. The PEDOT: PSS layer enhances the device's stability while also increasing its overall Seebeck coefficient.^[^ ^12^ ^]^ For comparative experiments, the metal layer is designed in two different shapes: T‐shaped and I‐shaped. The dimensions are as follows: for the T‐shape, L1 = 850 µm, L2 = 240 µm, W1 = 200 µm, W2 = 750 µm; for the I‐shape, L3 = 800 µm, L4 = 200 µm, L5 = 700 µm, W3 = 400 µm, W4 = 800 µm. Four units form one period. Based on the previous CST simulation experiments, this size design can effectively regulate the anisotropic polarization response of THz waves and generate thermoelectrically enhanced local fields, thereby effectively improving the sensitivity of THz detectors. The electron microscope images of the T‐shaped and I‐shaped PDs are shown in Figure 2B,D, clearly illustrating the periodic structure of the metasurface.

**Figure 3:**
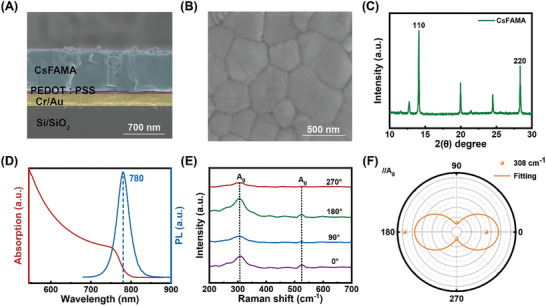
A) Cross‐sectional SEM image of CsFAMA/PEDOT:PSS PDs. B) SEM surface image of CsFAMA film. C) XRD pattern of CsFAMA film. D) PL spectra and absorption spectra within 500 to 900 nm wavelength. E) Angle‐dependent Raman spectroscopy at different polarization angles. (F) Polar plots of Raman modes Ag (308 cm^−1^).

**Figure** **3**A presents the cross‐sectional SEM image of the I‐shaped PDs, clearly showing the well‐ordered and distinct Au metasurface/PEDOT:PSS/CsFAMA layers. Figure S1 (Supporting Information) displays the metallographic microscopy of the metasurface layers, revealing a smooth surface with well‐maintained periodicity. The CST simulation results for the absorbance of I‐shaped PDs with different Au thicknesses (10, 100, 200, and 300 nm) are presented in Figure S2 (Supporting Information). The device exhibits the highest absorbance at an Au layer thickness of 200 nm. Figure S3 (Supporting Information) presents a cross‐sectional SEM image of CsFAMA/PEDOT:PSS film. Figure 3B shows the smoothness and uniformity of the perovskite film's surface, with no pinholes or cracks. Figure S4 (Supporting Information) shows the surface morphology and EDS spectrum of PEDOT: PSS on the metasurface metal layer. The PEDOT: PSS film shows a smooth surface with grains continuous and homogeneous uniformly. The EDS spectrogram of PEDOT: PSS presents the peaks of C, O, and S, which confirms the existence of PEDOT: PSS. The Atomic force microscopy (AFM) image of the perovskite film is presented in Figure S5 (Supporting Information). X‐ray diffraction (XRD) is shown in Figure 3C and Figure S6 (Supporting Information). The perovskite has good crystallinity, with diffraction peaks at 14.24° (110) and 28.58° (220) being characteristic peaks of the perovskite phase well reflecting the formation of perovskite. The peak at 12.6° corresponds to PbI_2_. To better understand the photophysical properties of perovskite thin films, photoluminescence (PL) and ultraviolet‐visible (UV–vis) spectroscopy analyses were performed (Figure 3D). The absorption spectra show a decreasing trend from ultraviolet to infrared, with a typical absorption peak at 780 nm. The perovskite film also exhibits good light absorption behavior, corresponding to the PL peak. Figures S7 and S8 (Supporting Information) illustrate the absorption and transmission spectra of CsFAMA in the 0–0.4 THz range. CsFAMA displays two distinct absorption peaks in this range, located at 0.04 THz and 0.1 THz, respectively. At 0.1 THz, the absorption of CsFAMA reaches a maximum of 33%. Figure 3E and Figure S9 (Supporting Information) display the Raman polarization spectrum of CsFAMA, revealing clear Ag modes at 308 and 526 cm^−1^, where resonant enhancement occurs.^[^ ^45^ ^]^ The intensity dependence on angle at these two points is illustrated in Figure 3F and Figure S10 (Supporting Information), showing significant variations with polarization angle. The intensity is strongest at 0°/180° and weakest at 90°/270°, with a fitted curve resembling a figure‐8 shape, consistent with the previously reported polarization pattern. The I‐shaped PDs exhibit strong anisotropy.

**Figure 4:**
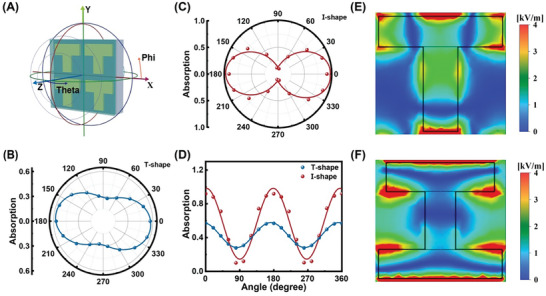
A) CST schematic diagram of polarized PDs. B,C) Polar coordinates angle‐dependent absorption under different polarized light irradiation of T‐shaped and I‐shaped PDs, respectively. D) Angle‐dependent absorption in different shapes. E,F) The electric field diagram for T‐shape and I‐shape at 0.1THz.

The simulation schematic of the device on the *XY*‐plane, conducted using CST MICROWAVE STUDIO (CST), is defined in **Figure** **4**A. The THz is directed from the positive *Z*‐axis towards the negative *Z*‐axis. The Phi parameter represents the polarization angle, set as Phi = 0° in the positive *Y*‐axis direction, while the Theta parameter corresponds to the vertical incidence angle. The absorption coefficient is calculated by *S_a_* = 1‐*S_r_*‐*S_t_*, where *S_r_* is the reflection coefficient and *S_t_* represents the transmission coefficient.^[^ ^28^ ^]^ Figure 4B,C shows the polar coordinates angle‐dependent absorption under different polarized light irradiation of T‐shaped and I‐shaped PDs, respectively. Both of the two PDs exhibit polarization absorption performance as “8” shape. In a rectangular coordinate, the absorption coefficient values exhibit a sine wave variation with the polarization angle from 0° to 360°, as shown in Figure 4D. The maximum absorption values occur at 0°/180° angles and the minimum value at 90°/270° for both devices. Additionally, I‐shaped PDs demonstrate higher absorption, reaching up to 0.9, and exhibit a higher anisotropy ratio compared to T‐shaped PDs.

The significant absorption can be attributed to the metasurface structure. THz SPP resonance within metasurfaces induces an enhanced local thermoelectric field, thereby enhancing PTE detection efficiency and THz response. Figure 4E,F and Figure S11 (Supporting Information) show the electric field intensity distributions with and without metasurface structure. Figure S11 (Supporting Information) presents the electric field profile without a metasurface structure, revealing the highest electric field of 1 kV m^−1^. In contrast, Figure 4E,F demonstrate that the metasurface structure enhances the electric field intensity to 4 kV m^−1^. This enhancement can be attributed to the excitation of SPP, which generates hot carriers within the structure. The interaction of these hot carriers with phonons produces significant heat.^[^ ^46^, ^47^ ^]^ In order to further explore the mechanism that I‐shaped absorption is better than T‐shaped absorption, the electric field intensity distributions of the T‐shape and I‐shape at *Z* = 0 are shown in Figure 4E,F, respectively. The electric field intensity in the I‐shaped PDs is significantly higher than in the T‐shaped PDs. Due to the SPP resonance effect of the metasurface, a stronger localized electric field is generated around the Au. The Electric field orientations of T‐shaped and I‐shaped PDs at 0.1THz are shown in Figure S12 (Supporting Information). Compared to the T‐shaped structure, the I‐shaped structure has more boundary constraints, causing less deviation from the main electric field direction and resulting in a stronger electric field and higher absorption.^[^ ^28^, ^48^, ^49^ ^]^

**Figure 5:**
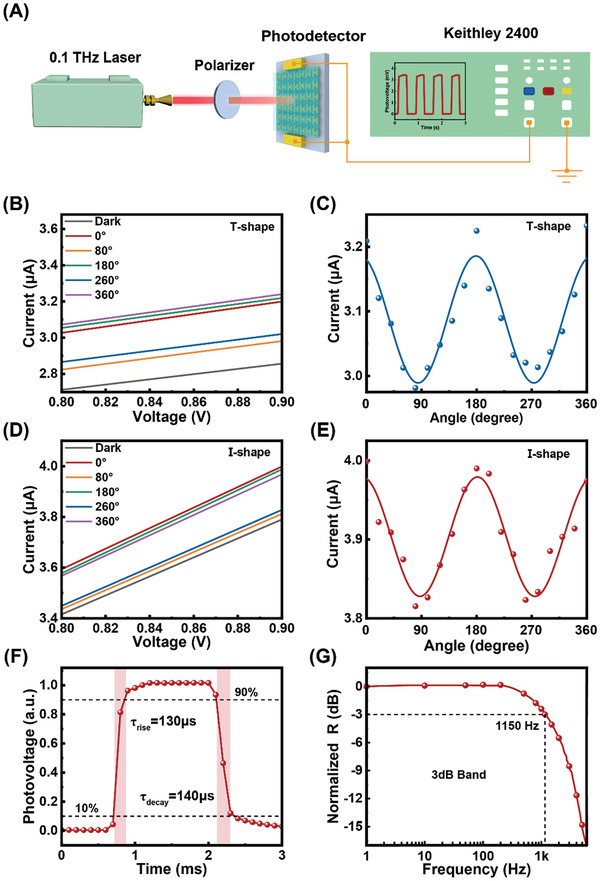
A) Schematic diagram of photovoltage response test system. B,D) *I–V* characteristics of PDs under dark and 0.1THz illumination. C,E) T‐shaped and I‐shaped PDs photocurrents corresponding to different polarization angles under 0.9 V bias voltage. F) Rise time and decay time of the photovoltage response. (I‐shape) G) Normalized response as a function of modulation frequency at −3dB.

The polarization optoelectronic response properties of the two PDs are illustrated in **Figure** **5**. The testing system, shown in Figure 5A, uses a 0.1THz laser source and a linear polarizer to obtain linearly polarized THz radiation. Figure 5B–E displays the current–voltage (*I*–*V*) characteristics of the T‐ and I‐shaped PDs under dark conditions and 0.1THz radiation at different polarization angles from 0° to 360°. The linear *I–V* curves within a small bias voltage range exhibit Ohmic contact, and their parallel variation with changing polarization indicates a typical PTE effect, as shown in Figure 5B,D. The *I–V* curves from −1 to 1 V bias voltage range are shown in Figures S13 and S14 (Supporting Information). The photocurrents increase with the increase of applied bias voltage. Figure 5C,E displays the photocurrents at different polarization angles of THz radiation at 0.9 V bias voltage. The I‐shaped PDs exhibit a higher photocurrent, ≈4.0 µA. The photocurrents exhibit an excellent polarization response characteristic, following a sine wave variation with the polarization angle range from 0° to 360°. The maximum values occur at 0°/180°/360°, while the minimum values occur at 90°/270°, consistent with the absorption patterns shown in Figure 4D. This behavior can be attributed to the anisotropy of the perovskite materials and the metasurface structures. Response time is a crucial performance parameter for PDs. Figure 5F displays the turn‐on/off light switch response of the I‐shaped PD, demonstrating a rise time of 130 µs and a decay time of 140 µs. The rise time is defined as the time for the photocurrent to increase from 10% to 90% of its maximum value, and the decay time is the time for the photocurrent to decrease from 90% to 10% of its maximum value.^[^ ^9^ ^]^ The response time can also be determined using the −3 dB bandwidth method, with the relationship given by: *t* = 1/2*πf*, where *f* is the −3 dB bandwidth frequency and *t* is the response time. Figure 5G shows the normalized *R* ‐3 dB bandwidth of the device under 0.1THz irradiation, and the corresponding response time is 138 µs.

**Figure 6:**
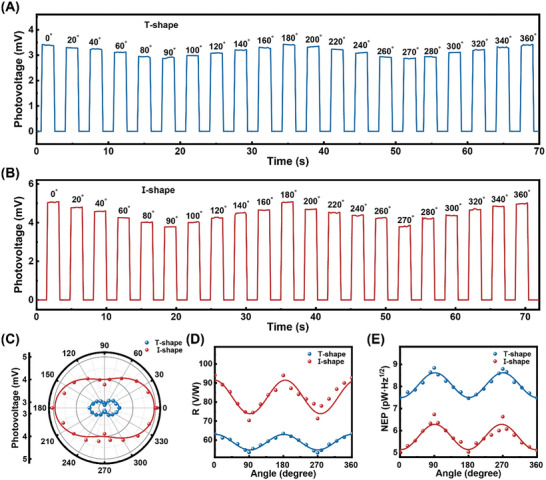
A,B) Photovoltage response of the different perovskite metasurface under 0.1THz laser incident at different polarization angles. The 0° polarization angle is defined as the positive direction of the *Y*‐axis. C) Polar plot of the photovoltage under different polarization angle illumination. D)*R* and E) *NEP* of the two PDs at different polarization angles.

**Figure** **6** shows the photovoltage on/ off optical switching responses of the CsFAMA PDs under 0.1THz laser irradiation at a 0.9 V bias voltage. The device exhibits repeatable and rapid on/off switching behavior across the 0–360° polarization range, as shown in Figure 6A,B. The maximum response occurs at 0°/180°, while the minimum occurs at 90°/ 270°. For T‐shaped PDs, the maximum photovoltage is 3.4 mV, and the minimum is 2.8 mV. Conversely, for I‐shaped PDs, the maximum is 5.1 mV, and the minimum is 3.7 mV, both following the polarization response pattern. Figure 6C illustrates the photovoltage response of PDs at different polarization angles in a polar coordinate system, where both the two devices exhibit a typical “8” shape. To evaluate the sensitivity of the PDs to linearly polarized light, the linear polarization anisotropy ratio (*g*) is introduced, representing the relationship between the maximum photoresponse and the minimum photoresponse, denoted as *g* = *I_max_*/*I_min_* = *V_max_*/*V_min_*. The calculated *g* values for the T‐shaped and I‐shaped PDs are 1.22 and 1.38, respectively. The polarization sensitivity and overall performance of these PDs are outstanding compared to previously reported results.^[^ ^14^, ^15^, ^16^ ^]^ The significant photovoltage polarization response is attributed to the SPP resonance and local thermoelectric field enhancement effect of the metasurface.^[^ ^50^ ^]^

Responsivity (*R*), detectivity (D^*^), and noise‐equivalent power (*NEP*) are the key parameters for evaluating the optoelectronic performance of detectors.^[^ ^51^ ^]^ *R* is the ability of photoelectric conversion, and the calculation formula is:

(1) $$R=\frac{\Delta V}{P}=\frac{V_{illu}-V_{dark}}{E_{e}\times A}$$

where *V_illu_* and *V_dark_* are the voltages under light and dark conditions, respectively. *P, Ee, e*, and *A* represent laser power, laser irradiance, electron charge, and effective illuminated area, respectively. D^*^ and *NEP* represent the ability for weak light detection, and the formula is:

(2) $$D^{*}=\frac{RA^{1/2}}{{\left(2eV_{dark}\right)}^{1/2}}$$

(3) $$NEP=\frac{A^{1/2}}{D^{*}}$$

According to formulas (1) and (2), *R, NEP*, and D^*^ at different polarization angles are shown in Figure 6D,E, and Figure S15 (Supporting Information). The maximum *R* and *NEP* of the T‐shaped PDs is 63.4 V/W and 7.46 pW/Hz^1/2^, with a maximum *D* ^*^ of 2.2 × 10^10^ Jones. Meanwhile, the I‐shaped PDs demonstrate a high *R* and low *NEP* of 94 V/W, 5.03pW/Hz^1/2^, and a high D^*^ of 3.28 × 10^10^ Jones. The cross‐sectional electric field distributions in Figure 4E,F reveal that the I‐shaped PDs generate stronger local electric fields, leading to higher absorption.^[^ ^52^ ^]^

**Figure 7:**
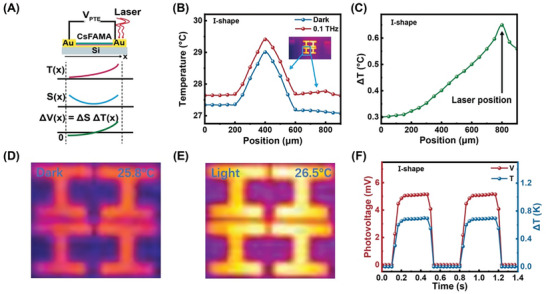
A) Schematic diagram of PTE effect. B) Temperature distribution diagram of the device under dark and 0.1THz laser irradiation of I‐shape device. C) Temperature rise curve of the device under 0.1THz illumination I‐shape. D,E) Temperature distribution of I‐shaped device under dark and 0.1THz laser irradiation. F) Photovoltage (red line) and temperature (blue line) turn on/off curves under 0.1THz light illumination at 0.9 V bias voltage I‐shape device.

The photoresponses mechanism of the devices under THz illumination is thoroughly discussed. Based on the previous studies,^[^ ^10^, ^11^, ^12^ ^]^ photovoltage (PV) or photoconductive (PC) effects are limited to the THz detection wavelength range where the bandgap of perovskite materials is larger than the photon energy of THz radiation. In such scenarios, thermoelectric effects may play a significant role. The *I–V* curves in Figure 5B,D exhibit typical photothermoelectric (PTE) effect.^[^ ^12^ ^]^ The PTE effect mechanism diagram can be expressed as **Figure** **7**A, setting the *X*‐axis along the channel. *ΔS* represents the Seebeck gradient along the positive *X*‐axis, while *ΔT* denotes the temperature gradient in the same direction. When THz illumination strikes one electrode, it causes a local temperature increase and a redistribution of temperature in the irradiated region. Hot carriers generated by thermal excitation accumulate in regions of higher temperature. Additionally, the interface between two materials with different Seebeck coefficients induces a Seebeck gradient (*ΔS*), which drives the diffusion of hot carriers along the temperature gradient. Under these conditions, an electrical potential gradient *V_PTE_* is created.^[^ ^53^ ^]^ The formula for calculating PTE voltage is given by:

(4) $$V_{PTE}=\Delta S*\Delta T$$

Here, *S* is the Seebeck coefficient, *T* is temperature and *∆* represents the gradient. For PTE effect, the dynamics of hot carriers typically dominate the generation of photocurrent due to the inefficient cooling of lattice electrons.^[^ ^11^, ^54^, ^55^ ^]^ Schematic of the device band energy under dark and 0.1 THz illumination as shown in Figure S16 (Supporting Information). The heterojunction formed by CsFAMA/PEDOT: PSS, influenced by both the internal electric field and the PTE electric field, promotes the transport and separation of thermal carriers, thereby significantly enhancing the device's detection performance.

To further confirm the effectiveness of PTE, we conducted the following experimental validation on I‐shaped PDs using an infrared thermal imaging camera shown in Figure 7B–F. The temperature distribution of the device was measured using an infrared thermal imager (FLIR T420) in conjunction with a macro lens. Temperature variation of the device is presented in Figure 7B, with the inset highlighting the active area. During the active phase, the blue line presents the temperature variation of the device in the dark, where the two electrodes remain consistent. When THz irradiation is applied to one end of the device, the temperature gradient in the active region changes significantly, as indicated by the red curve. Notably, the temperature rise at the THz exposure end is higher than at the non‐exposure end, with a temperature peak ≈0.65 °C shown in Figure 7C. Under dark conditions, the CsFAMA/metasurface device shows a relatively low‐temperature of 25.8 °C. When illuminated by a 0.1THz laser with a power of 1.55 mW, the surface temperature increased by 0.7 °C, as shown in Figure 7D,E, respectively. Furthermore, Figure 7F exhibits the photovoltage and temperature‐switching curves. It can be seen that the photocurrent and temperature reveal the same tendency under THz illustrated. This confirms that the generation of photocurrents in the CsFAMA/ metasurface device depends on the PTE effect.

**Figure 8:**
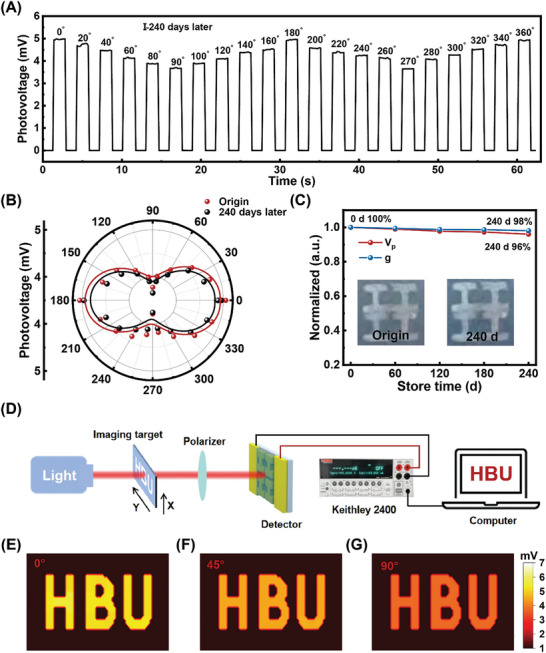
A) Photovoltage response of I‐shaped PDs tested after storage in air condition for 240 days. B) Polar plot of the photovoltage at different polarization angles. C) Photovoltage and polarization ratio of I‐shape PDs after storage in air condition for different times. D) Schematic of the test setup for polarization imaging. E–G) Imaging patterns of “HBU” under 0.1THz light at 0 deg (left) 45 deg (middle) and 90 deg (right).

Currently, the stability of perovskite devices is a feature of concern. Therefore, we conducted a stability test on the I‐shaped PDs by exposing the device under air conditions for 240 days. The polarization response characteristics of the device under 0.1THz illumination were tested every 60 days to evaluate the stability as shown in **Figure** **8**. Figure S17 (Supporting Information) and Figure 8A demonstrate stable and consistent photovoltage turn‐on/off switching characteristics after 60, 120, 180, and 240 days under the same 0.1THz radiation. The CsFAMA/metasurface device also shows excellent stability. Comparisons of the I‐type device's photovoltage comparison in polar and linear coordinate systems at different time intervals are explored and shown in Figure 8B,C, and Figures S18 and S19 (Supporting Information). After 240 days, the device still exhibited excellent “8” type performance, with a maximum photovoltage of 4.9 mV and a minimum photovoltage of 3.6 mV, indicating only a 4% performance decrease. Moreover, the polarization ratio decreased from 1.38 to 1.36, a reduction of 2%. Furthermore, the insert pictures in Figure 8C show no significant change in the surface of the device before and after exposure to air conditioning for 240 days. These results confirm the good stability of the PDs. Accelerated aging tests under high‐temperature and humidity were conducted on both I‐shaped and T‐shaped PDs, as shown in Figures S20–S27 (Supporting Information). The results demonstrate that the devices exhibit excellent reliability and durability in practical applications.

In addition, utilizing the excellent polarization response and stability, the CsFAMA/metasurface PDs were also evaluated for imaging applications. To demonstrate the polarization imaging capability of the I‐shaped device, a single‐point pixel detection imaging experiment was conducted after the device had been exposed to an air environment for 240 days. As shown in Figure 8D, a hollow mask with the “HBU” is designed and controlled by a three‐position mobile platform. The laser passes through the hollow mask and polarizer sequentially before irradiating the device. The position of the hollow mask and controlled by the 3D mobile platform, and the photovoltage is recorded in real‐time, corresponding to the mask position. Figure 8E–G illustrates the different polarization photovoltage imaging results at 0° (left), 45° (middle), and 90° (right) polarization angles under 0.1THz illumination. High‐resolution images of “HBU” with clear borders are presented with a maximum photovoltage ratio of 5.1 at 0°, and a minimum of 3.7 at a polarization angle of 90°. Other shapes polarization imaging tests of “↑” and “*” shapes were performed, as shown in Figure S28 (Supporting Information). The results demonstrate clear and distinct polarization images for each shape, confirming that the device exhibits stable and excellent polarization imaging capabilities across different shapes. Table S1 (Supporting Information) shows a performance comparison of typical PDs. In a comprehensive evaluation, our devices demonstrate competitive performance in several aspects, including *g*, *R*, and *NEP*. The results are consistent with the polarization photoresponse shown in Figures 5 and 6.

## 3 Conclusion

In summary, we have demonstrated two distinct structures of perovskite metasurface PDs (T‐shaped and I‐shaped), showcasing polarization‐sensitive detection capabilities within the THz band. Under 0.1THz laser illumination, I‐shaped PDs exhibit enhanced local electric fields within their resonant cavity, facilitating sustained oscillation of internal high‐frequency electromagnetic fields. This results in superior performance, evidenced by a device responsivity reaching 94 V/W, noise equivalent power as low as 5.03 pW/Hz^1/2^, and an anisotropy ratio of 1.38. Moreover, a response time of 138 µs is achieved at room‐temperature. Notably, the device's performance shows minimal degradation, with only a 2% decrease after 240 days of exposure to air conditions. Furthermore, polarized imaging applications under 0.1THz laser illumination have been demonstrated. The synergistic combination of perovskite with metasurfaces not only enhances thermoelectric performance but also strengthens polarization capabilities, effectively overcoming the constraints imposed by traditional semiconductor materials. Metasurfaces pave the way for capturing multidimensional information, thereby expanding the application spectrum of the THz band. As this research progresses, the prospects for THz polarization detection appear increasingly promising. In the future, PDs utilizing the thermoelectric effect are expected to find broader applications across materials science, communication technology, medical imaging, and beyond, presenting new opportunities for scientific research for scientific inquiry and engineering advancement.

## Conflict of Interest

The authors declare no conflict of interest.

## Supporting information



Supporting Information

## Data Availability

The data that support the findings of this study are available from the corresponding author upon reasonable request.
