# Supplementary material for: A Room‐Temperature Terahertz Photodetector Imaging with High Stability and Polarization‐Sensitive Based on Perovskite/Metasurface
Source: Adv Sci (Weinh). 2024 Dec 17;12(6):2407634. doi: 10.1002/advs.202407634 (PMC11809364; doi:10.1002/advs.202407634)
Supplement: Supplementary file 1 — Supporting Information [file ADVS-12-2407634-s001.docx]

Supporting Information

**A Room Temperature Terahertz Photodetector Imaging with High Stability and Polarization-Sensitive Based on Perovskite/Metasurface**

Yifan Li, Yiming Jia, He Yang, Yinghui Wu^*^,Yajun Cao, Xuyang Zhang, Cunguang Lou, Xiuling Liu^*^ ,Long-Biao Huang^*^,and Jianquan Yao

Experimental Section

*Synthesis of the CsFAMA*

First, the indium tin oxide (ITO) glass in ethanol, acetone, and isopropanol for 15 min. After drying with condensed air, subjected it to UV-ozone treatment for 15 min. Next, prepared the mixed precursor solution of Cs_0.05_(FA_0.85_MA_0.15_)_0.95_Pb(I_0.85_Br_0.15_)_3_ by dissolving 1.02 M FAI, 0.18 M MABr, 1.105 M PbI_2_, and 0.195 M PbBr_2_ in 1 mL of DMF/DMSO (4:1) mixed solvent. Stirred the solution at 70°C for 1 hour to ensure complete dissolution. The following steps should be performed in a glove box. Spin-coated 50 ul of the perovskite precursor onto the substrate at speeds of 2000 rpm and 6000 rpm for 10 s and 30 s, respectively. During the final 10 s of the second step, slowly drop 300 ul of chlorobenzene (CB) onto the perovskite film as an anti-solvent. Finally, annealed the deposited perovskite thin film at 120°C for 20 min.

The use of Formamidinium iodide (FAI) and Methylammonium bromide (MABr) from Xi'an Polymer Light Technology Corp., along with Cesium iodide (CsI), Lead iodide (PbI_2_) and Lead bromide (PbBr_2_,) from Tokyo Chemical Industry. Unless otherwise specified, all solvents used in the experiments were obtained from Sigma Aldrich or Alfa Aesar.

*Device Simulation*

Devices were simulated using the Microwave module of CST Microwave Studio. The parameters of the device (PDs) are set in CST, utilizing a frequency domain solver. The frequency unit is GHz, with a frequency band ranging from 90GHz to 130GHz, and the dimensions are in micrometers (μm).

*Characterization and testing*

Using the MERLIN scanning electron microscope (SEM) from Carl Zeiss AG, Germany, operated at an electron energy of 5 kV, cross-sectional and top-down SEM images of the sample were obtained. Energy Dispersive Spectrometer (EDS) images were obtained by X-ray spectrometer. Atomic force microscopy (AFM) experiments were performed using an MFP-3D-BIO AFM (Asylum Research, Goleta, CA, USA). X-ray diffraction (XRD) patterns were performed using a multifunctional powder diffractometer (BRUKER ECO D8 1 KW). Additionally, Photoluminescence (PL) spectra of the sample were obtained by irradiating it with a 374 nm laser. Measured the absorption spectra using a UV/Visible/Near-Infrared spectrophotometer (LAMBDA 950, PerkinElmer, Waltham, MA, USA). Polarized Raman spectra of the sample were acquired using a Raman spectrometer from Renishaw, London, UK. The metasurface periodic structures of the device were identified by means of a metallographic microscope (CX40M). The *I-V* characteristics and photoelectric voltage response of the device were tested using a Keithley 2400 source meter and its virtual software. A 0.1 THz laser (THz-IMPATT) was used as the light source. Optical power was measured using a power meter with an RM9-THz Ophir port. Temperature distribution curves were obtained using an infrared thermal imaging camera (FLIR T420).


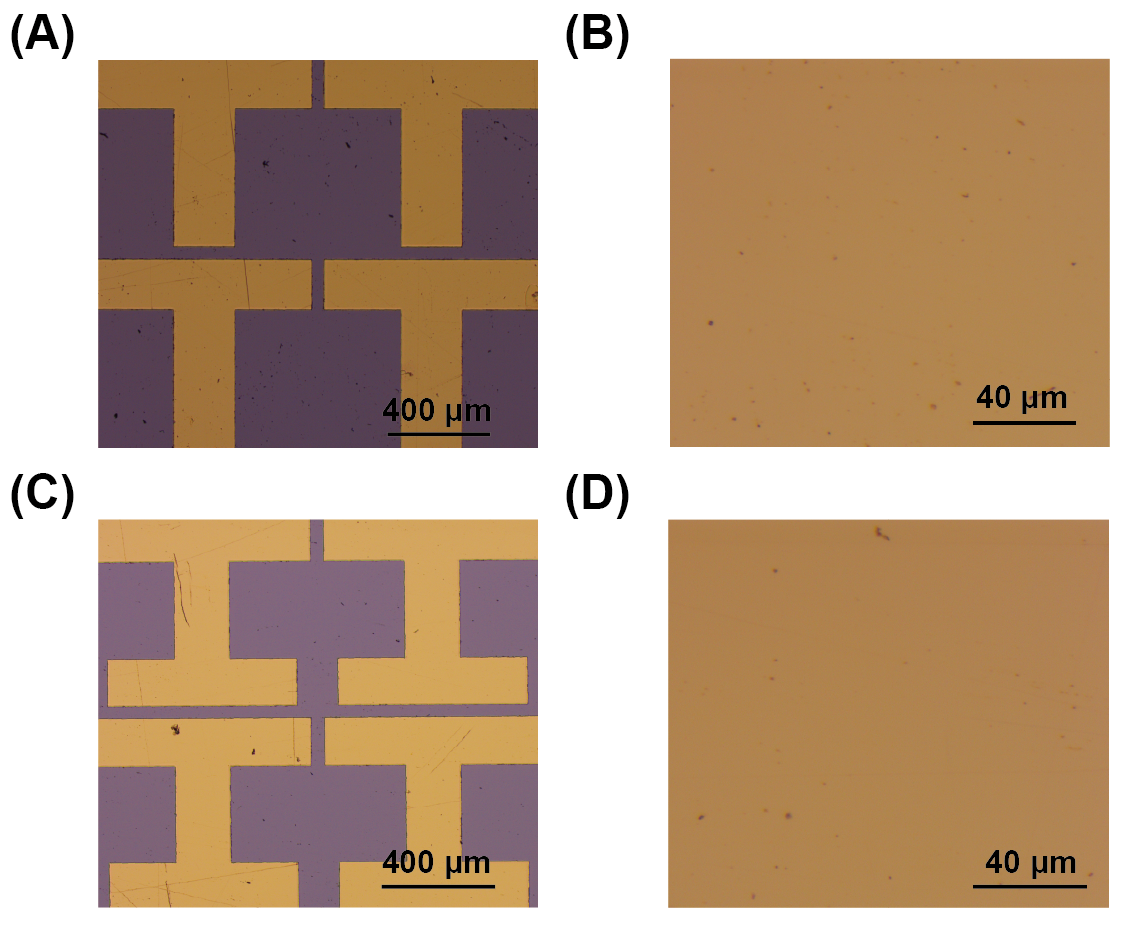


**Figure S1.** (A, B) Metallographic microscope image of metal layer (T-shaped PDs). (C, D) Metallographic microscope image of metal layer (I-shaped PDs).


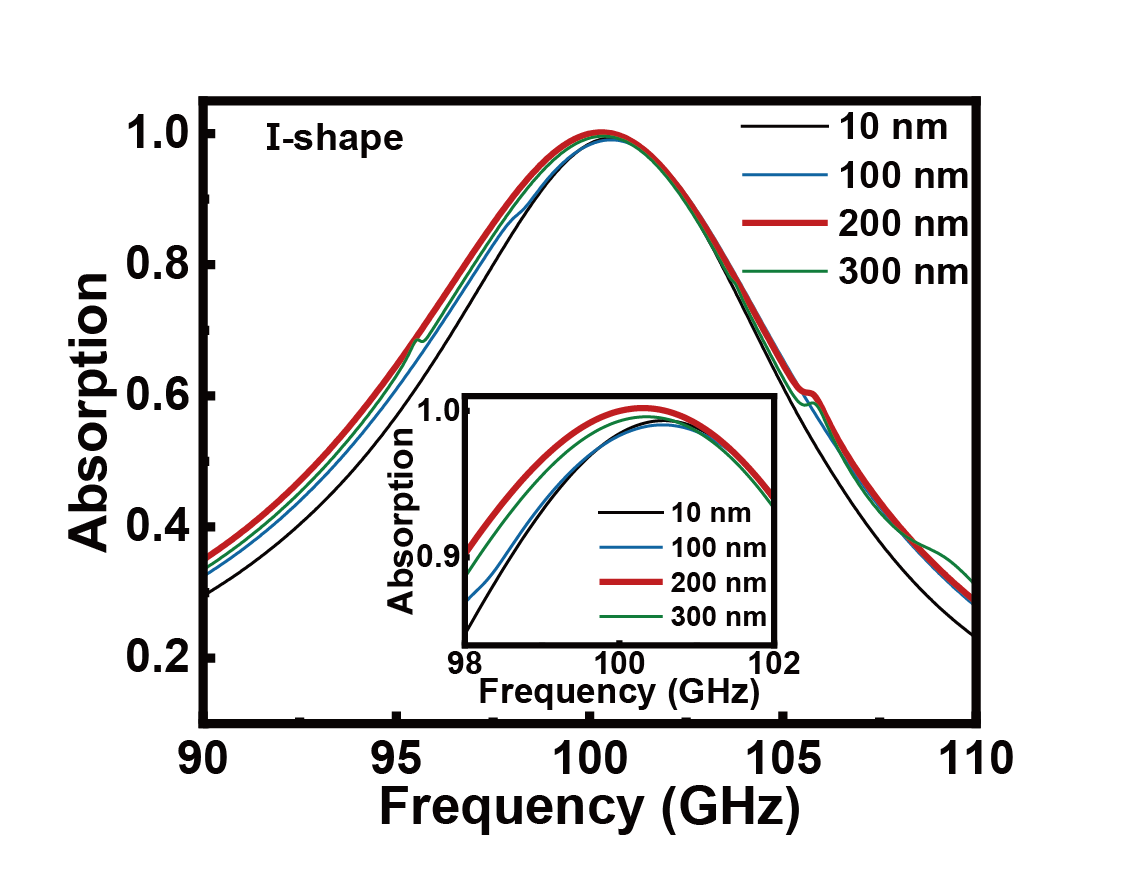


**Figure S2.** The absorption of I-shaped PD at different gold thickness.


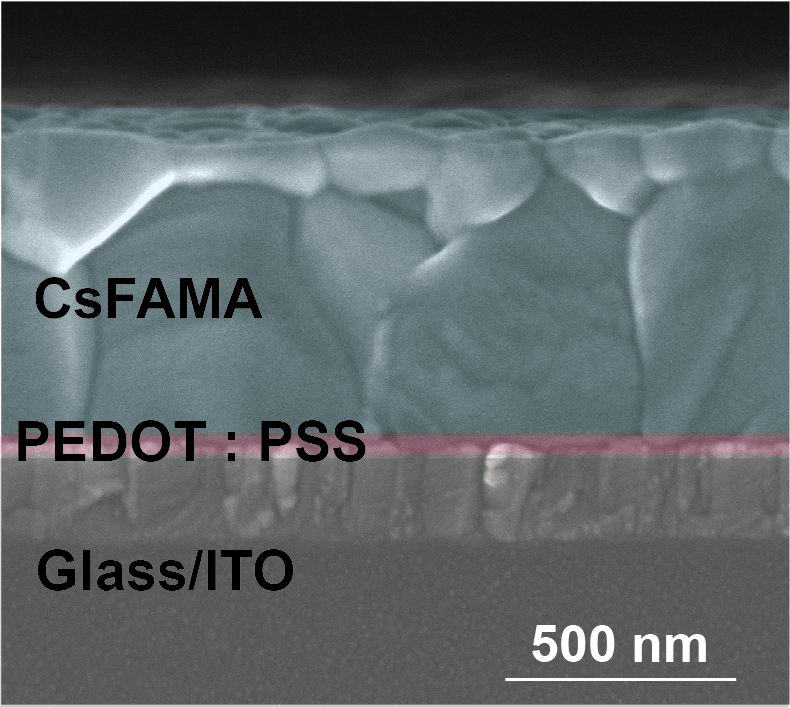


**Figure S3.** Cross-sectional SEM image of CsFAMA/PEDOT:PSS film.


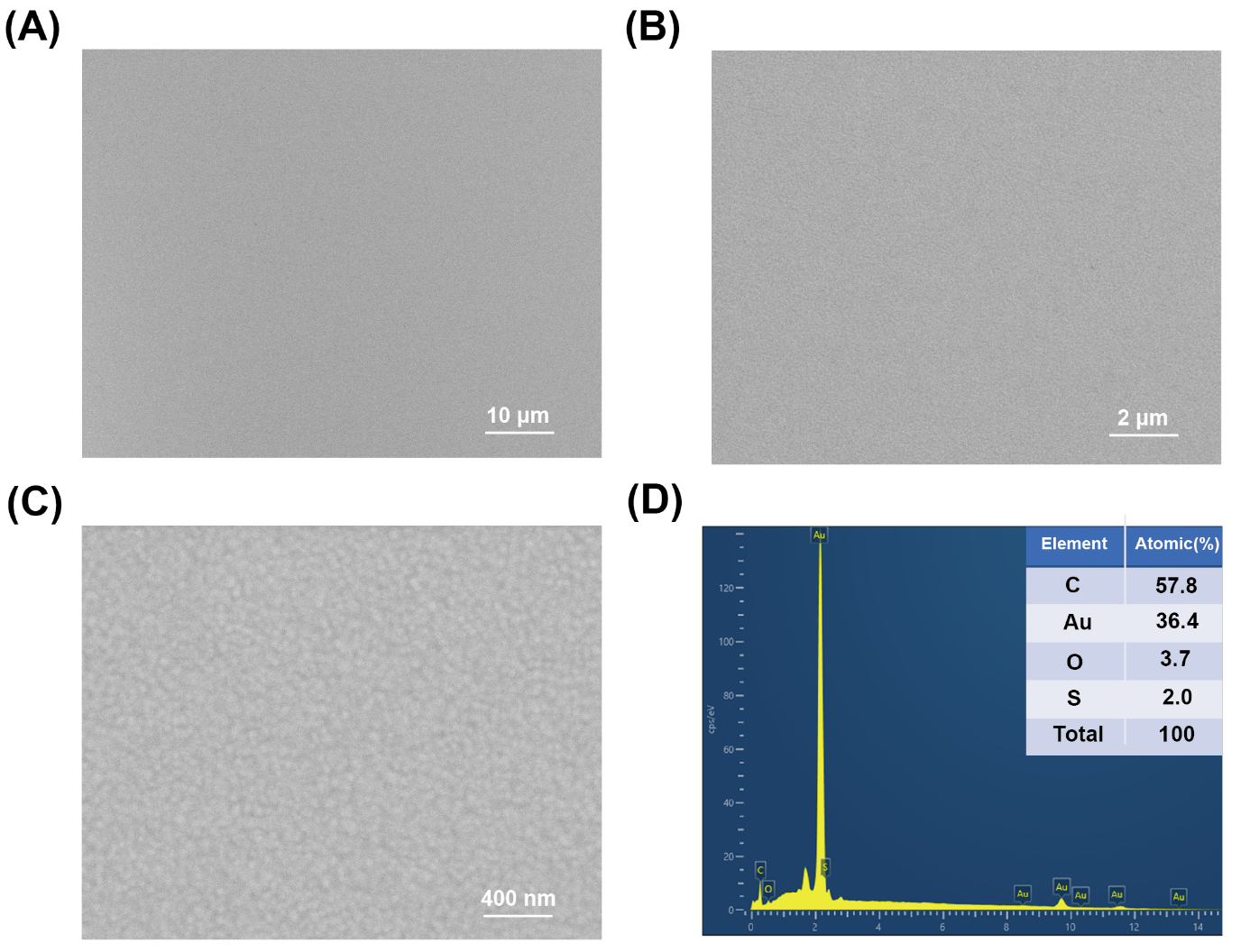


**Figure S4.** (A-C) SEM surface image of PEDOT:PSS ﬁlm. (D) Energy Dispersive Spectrometer (EDS) spectrum of PEDOT:PSS ﬁlm.


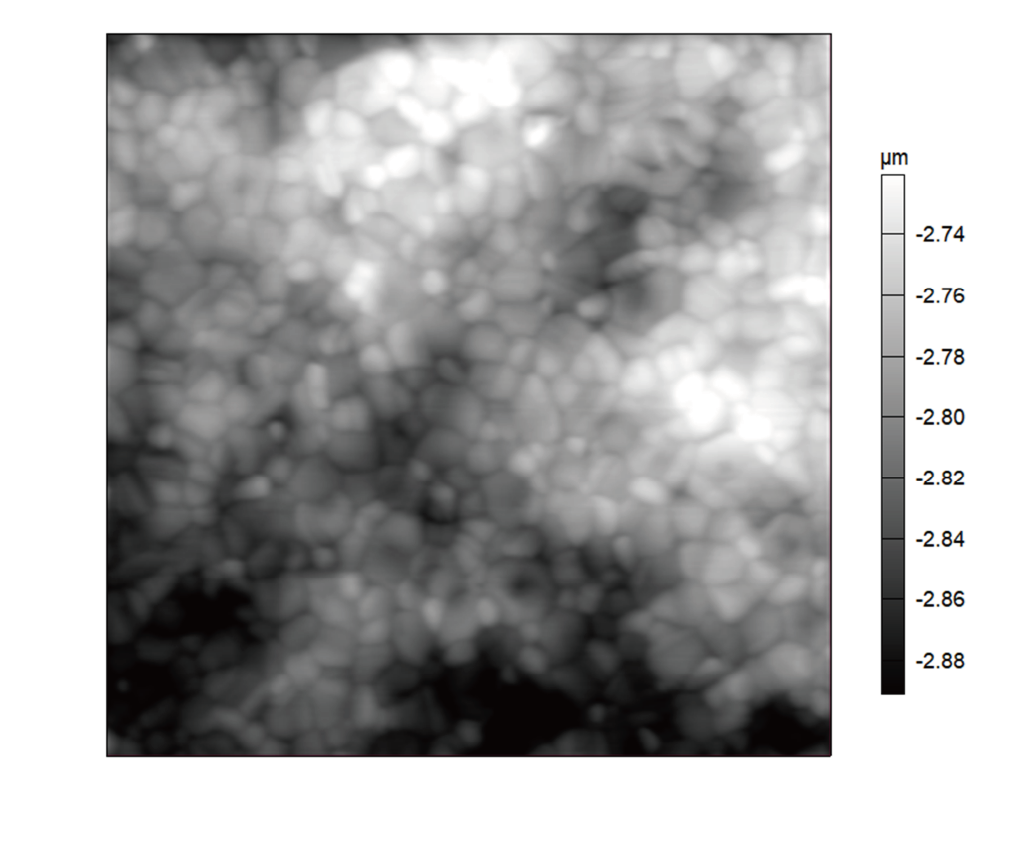


**Figure S5.** Atomic force microscopy (AFM) image of CsFAMA film.


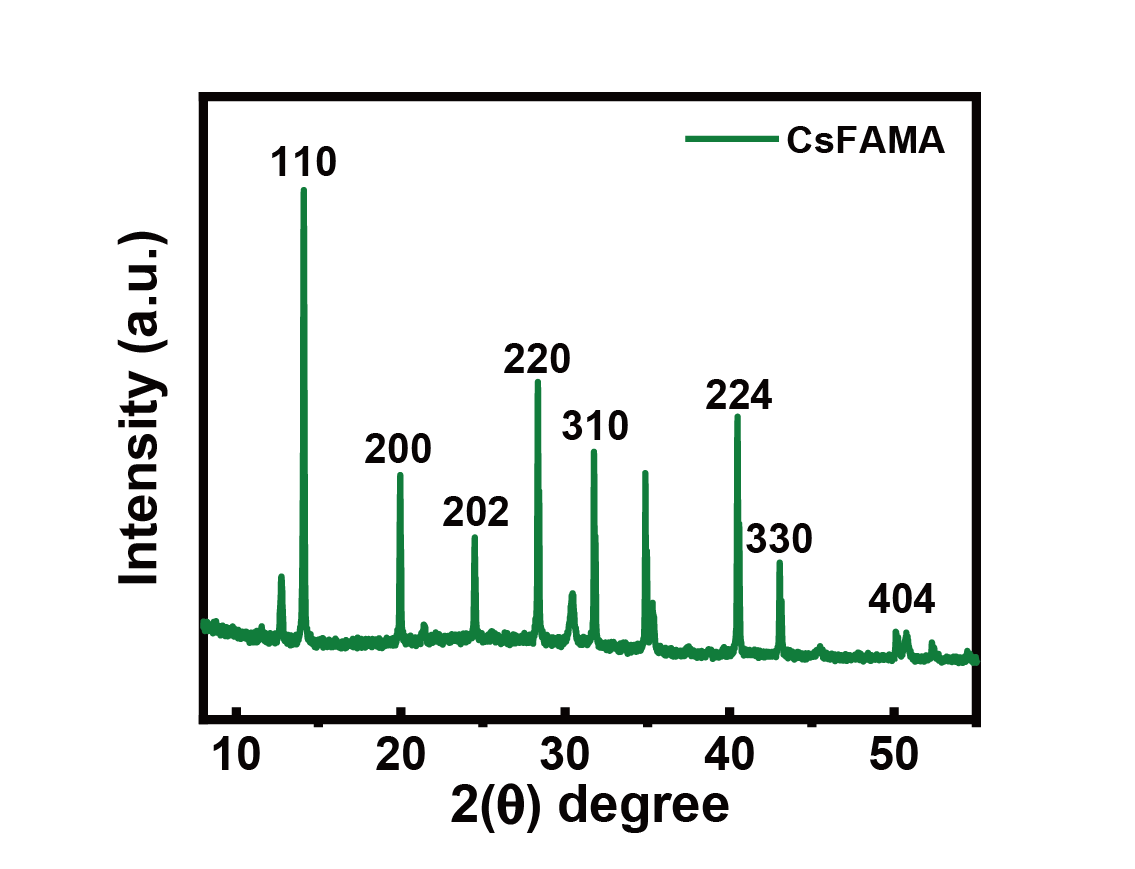


**Figure S6.** X-ray diffraction (XRD) pattern of the CsFAMA film.





**Figure S7.** THz absorption spectrum of CsFAMA.





**Figure S8.** THz transmission spectrum of CsFAMA.


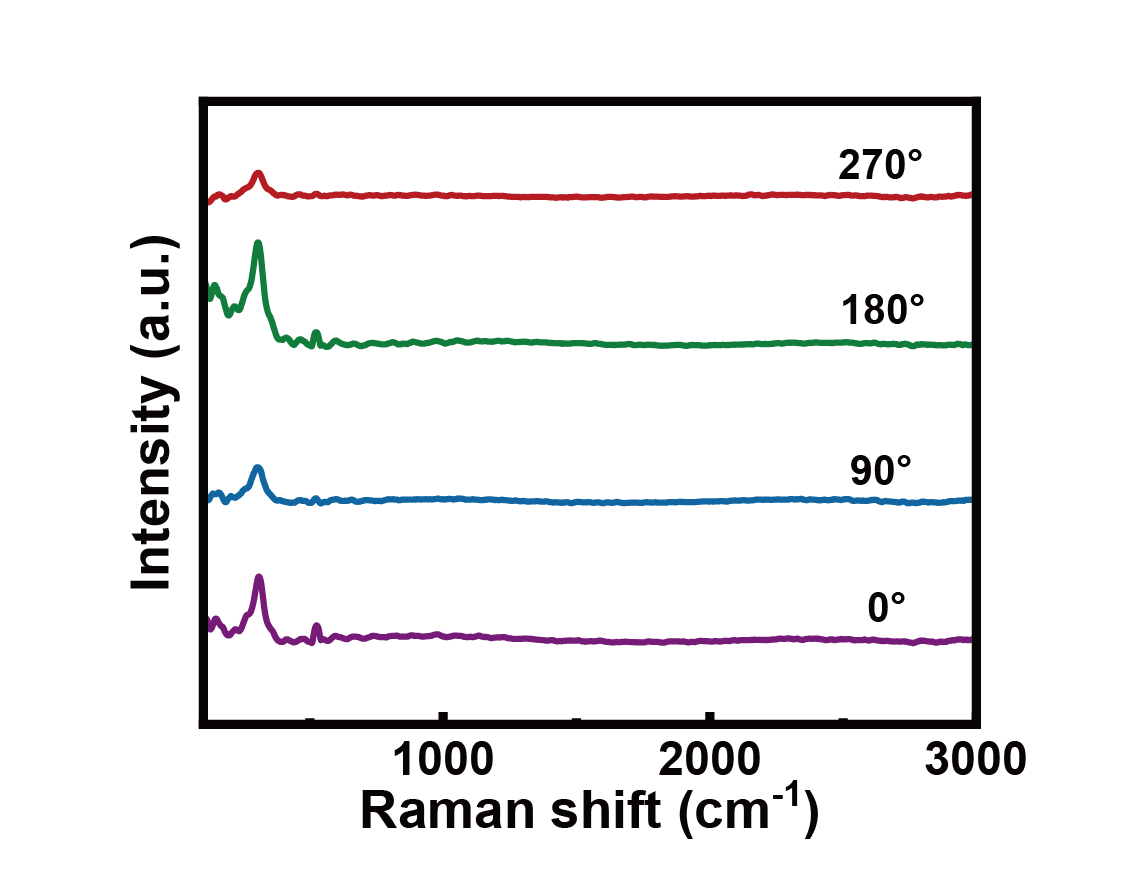


**Figure S9.** The polarized Raman of CsFAMA film.


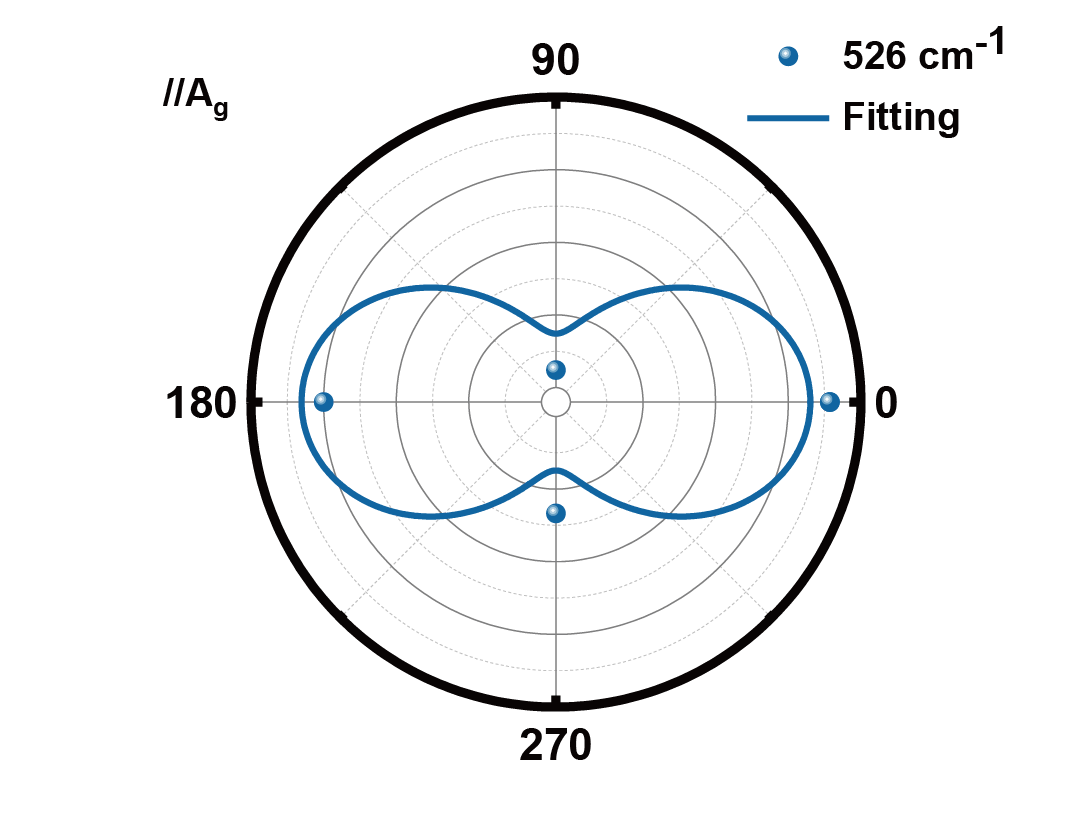


**Figure S10.** Polar coordinate diagram corresponding to the Raman peak intensity at 526 cm⁻¹.


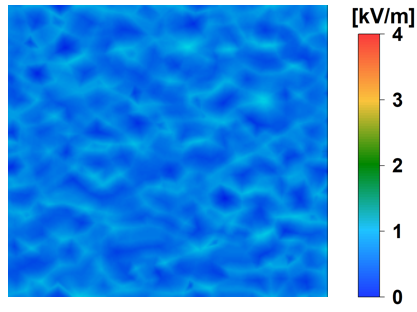


**Figure S11.** Electric field simulation without metasurface.


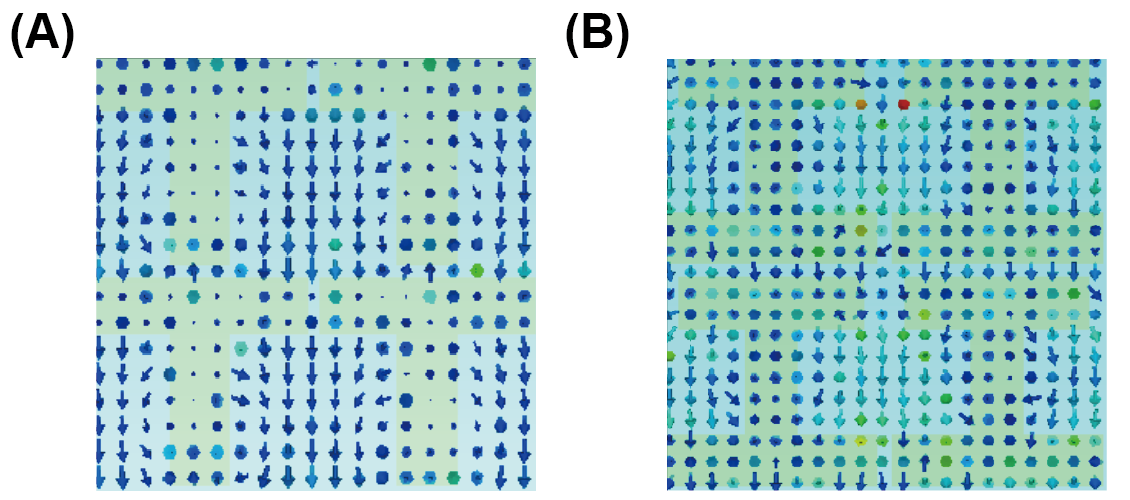


**Figure S12.** (A, B) Electric field orientation of T-shaped and I-shaped PDs at 0.1 THz T-shape and I shape, respectively.


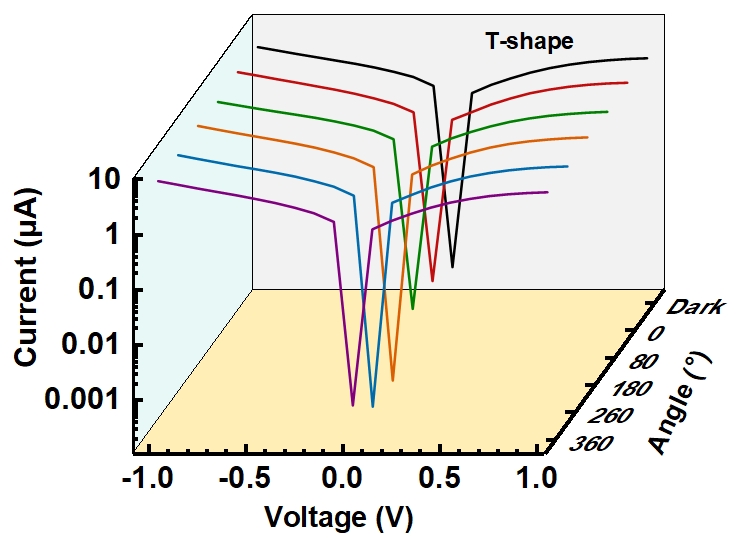


**Figure S13.** T-shaped *I-V* characteristic curve (-1V to 1V).


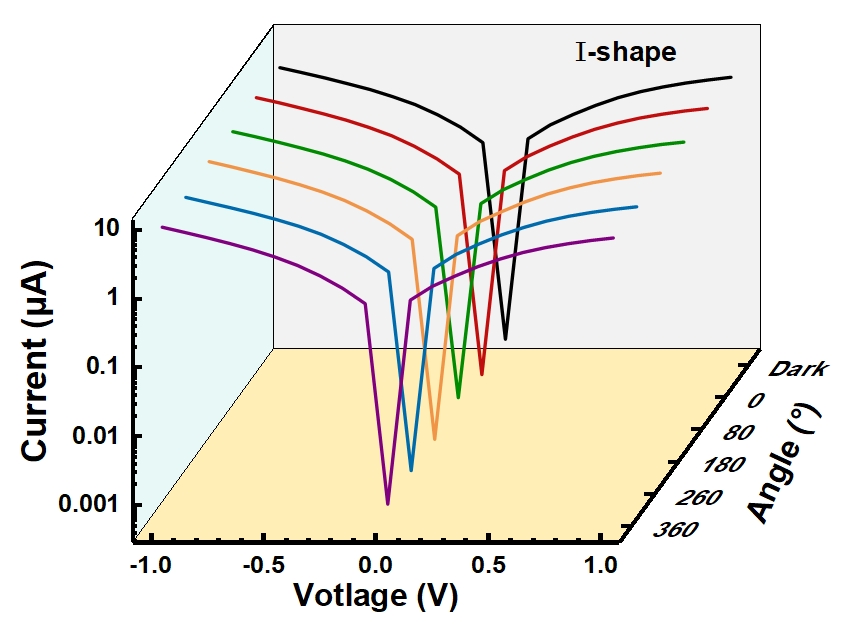


**Figure S14.** I-shaped *I-V* characteristic curve (-1V to 1V).


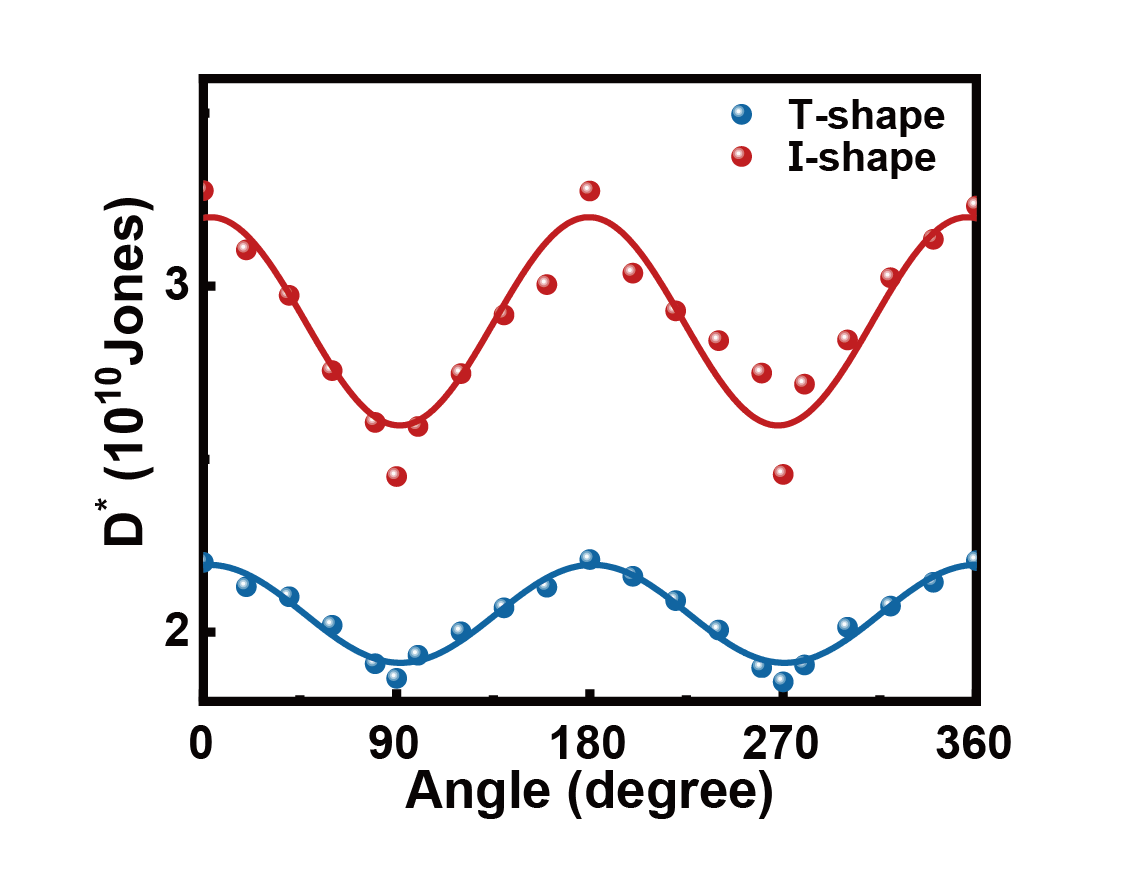


**Figure S15.** Normalized detection rate(*D^*^*) at different angles.

**
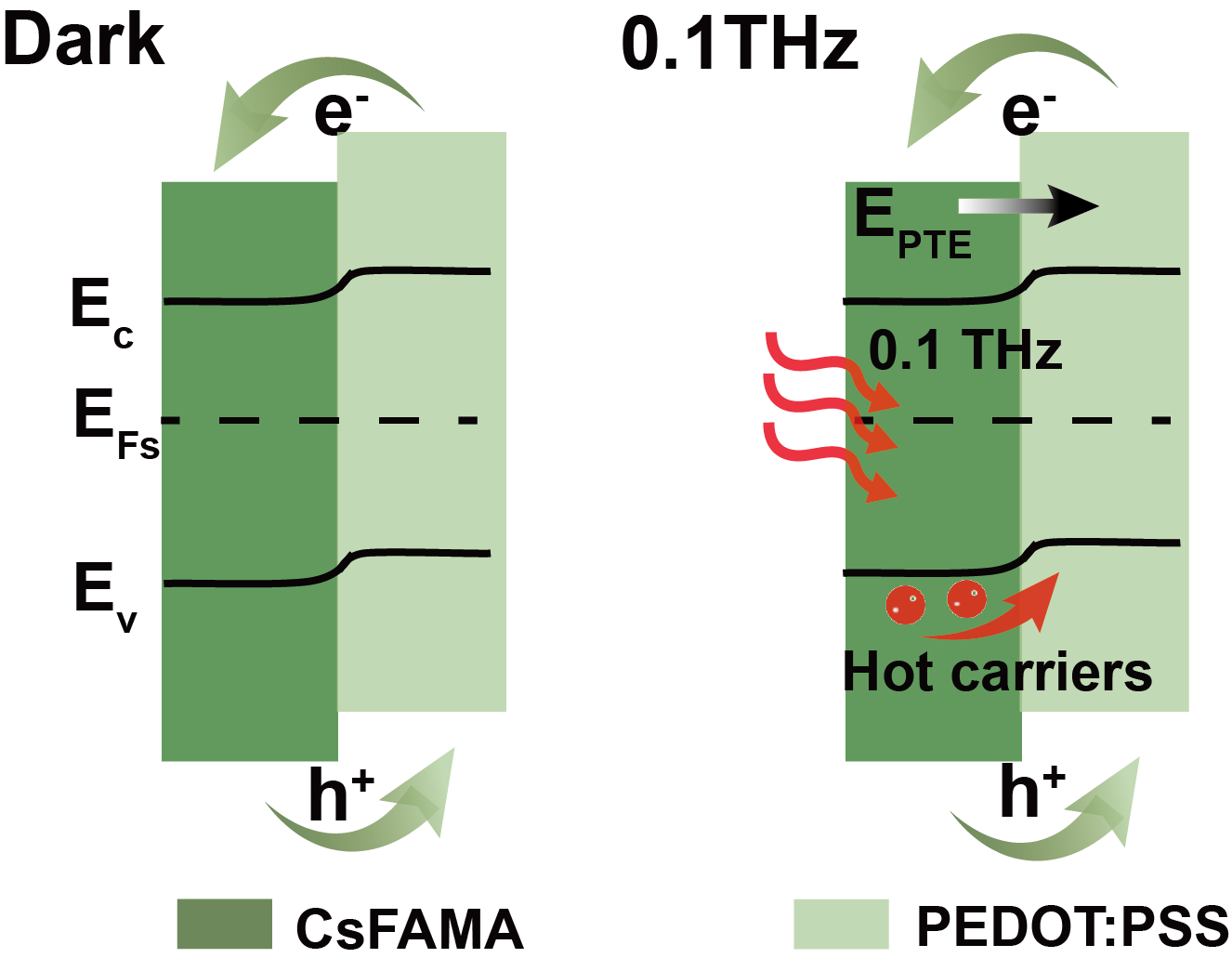
**

**Figure S16.** Schematic of the device band energy under dark and 0.1 THz illumination.


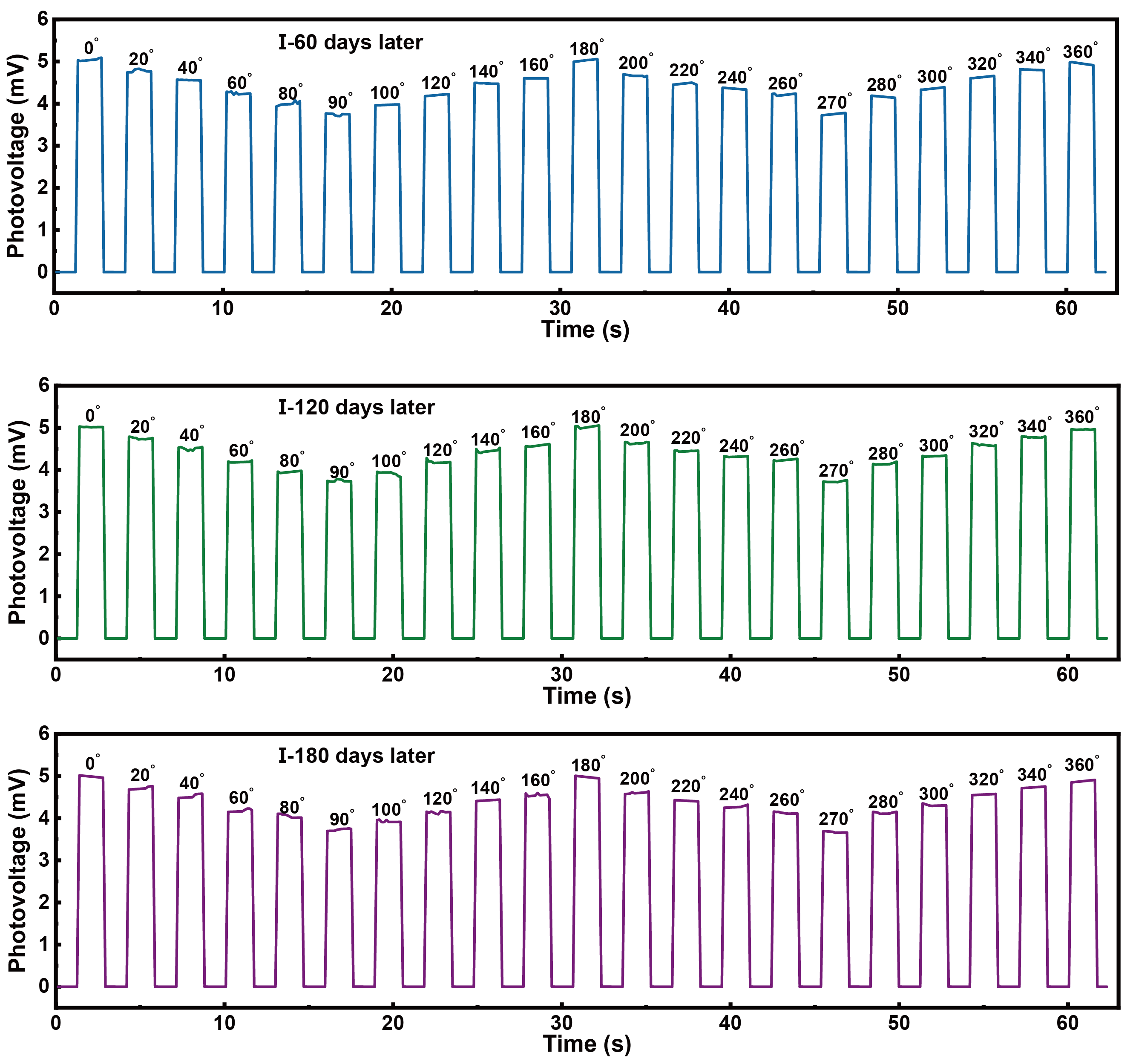


**Figure S17.** The photovoltage of I-shaped PDs measured after being placed in air condition for 60 days, 120 days, and 180 days.


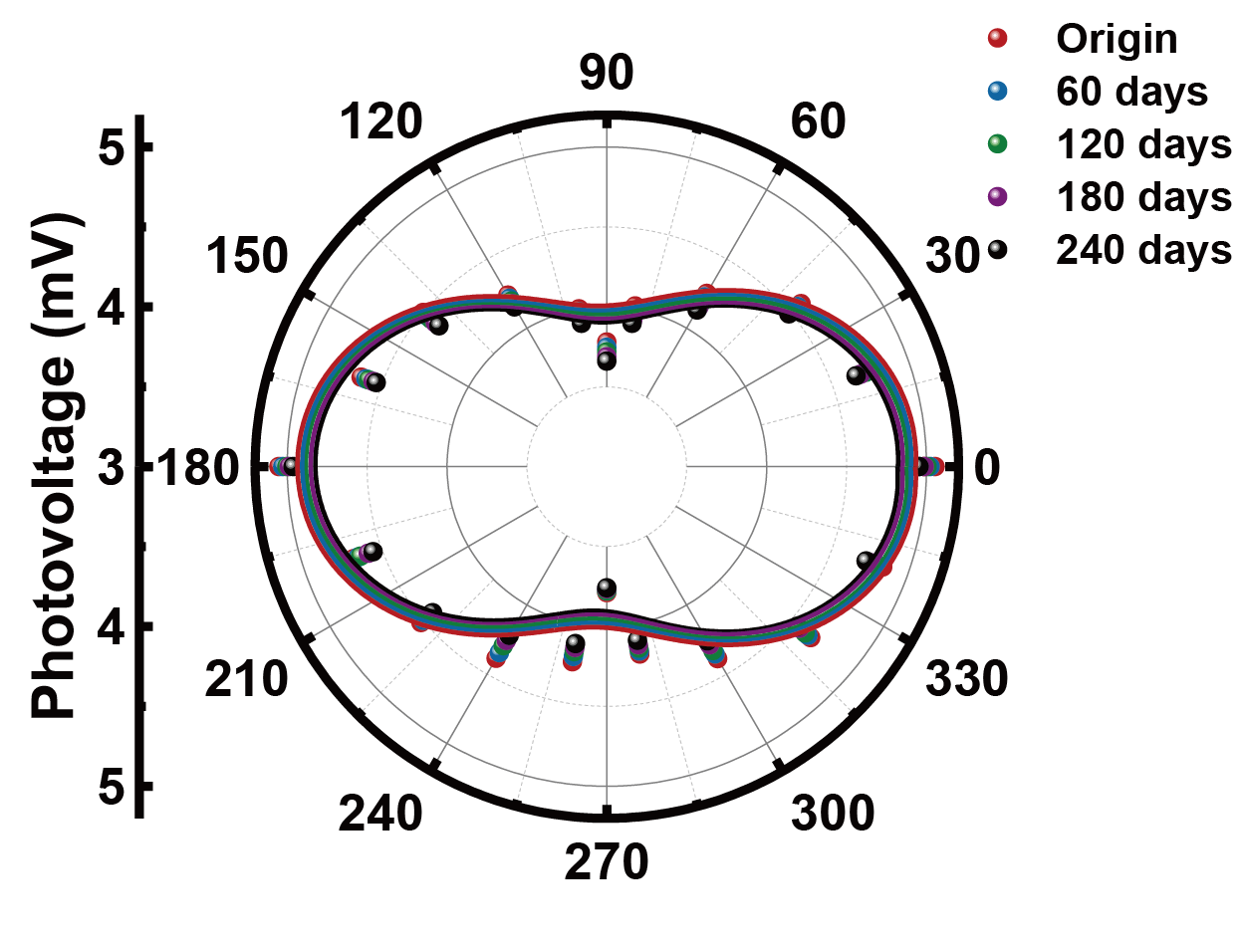


**Figure S18.** Polar plot of the photovoltage of I-shaped PDs at different time intervals.


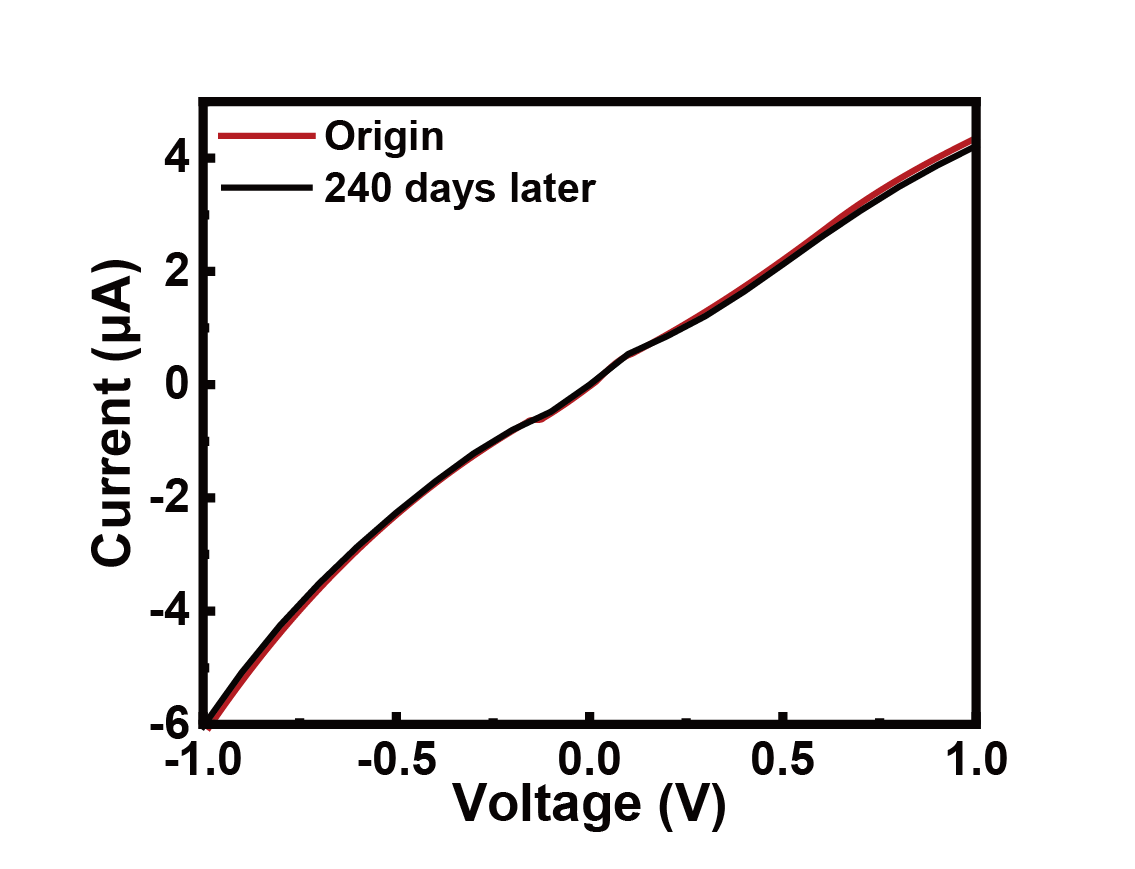


**Figure S19.** *I-V* characteristic curves of I-shaped PDs at the origin and after 240 days.


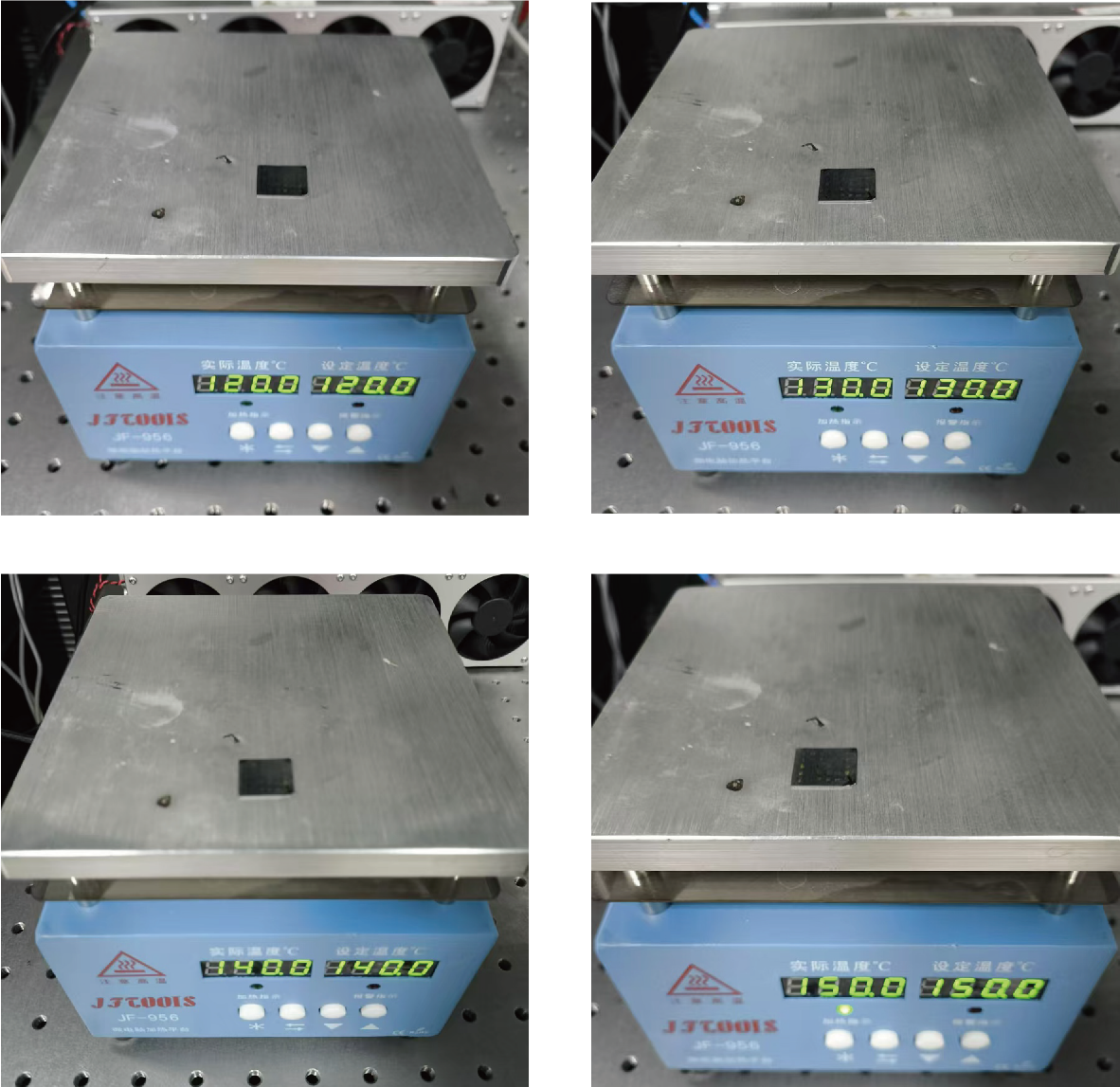


**Figure S20.** Heating diagram at different temperatures.


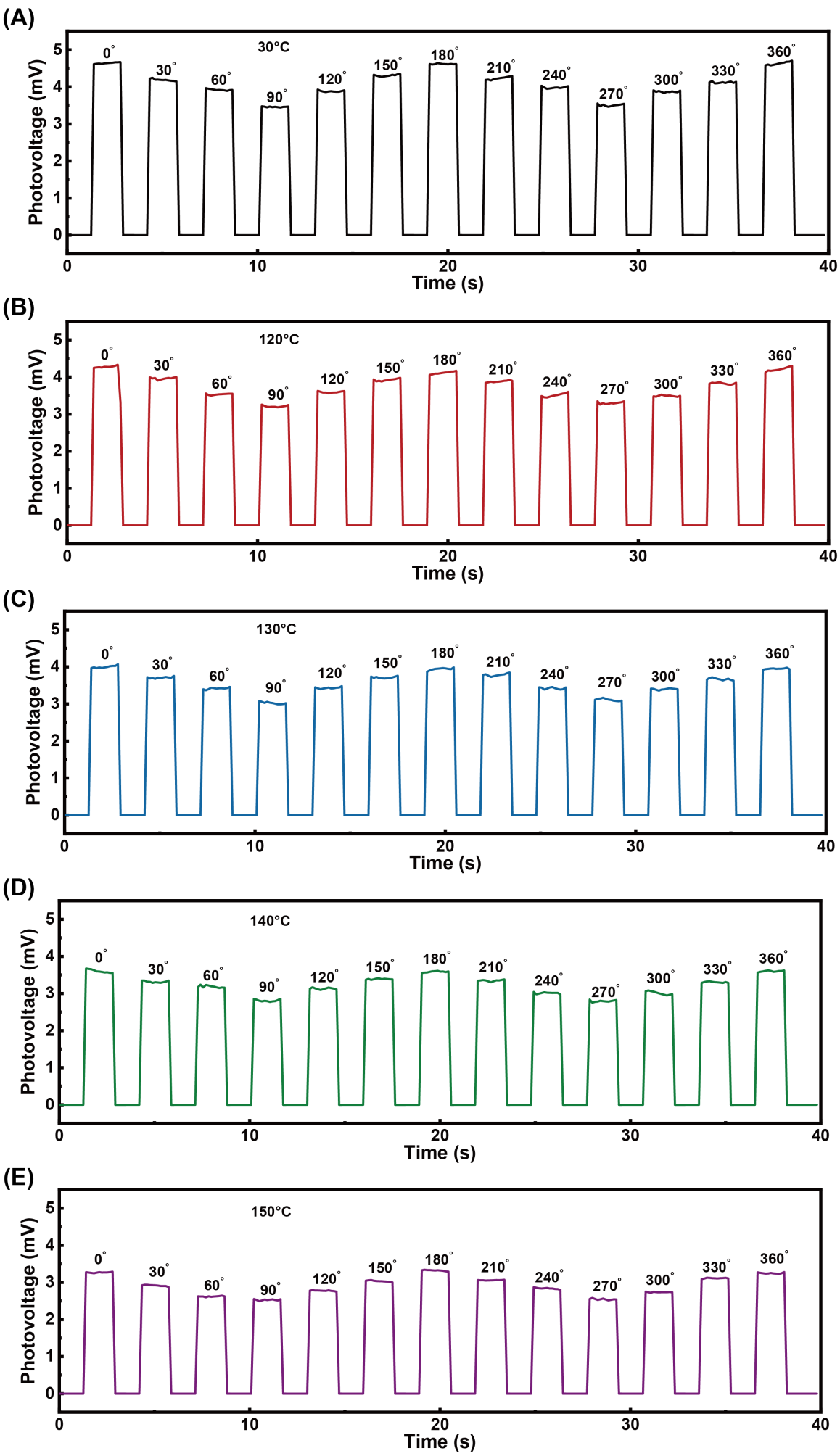


**Figure S21.** (A) The photovoltage of I-shaped PDs measured at room temperature after being placed in air condition for 360 days. (B) The photovoltage of I-shaped PDs measured after one hour of heating on a 120℃, 130℃, 140℃, and 150℃ heater.


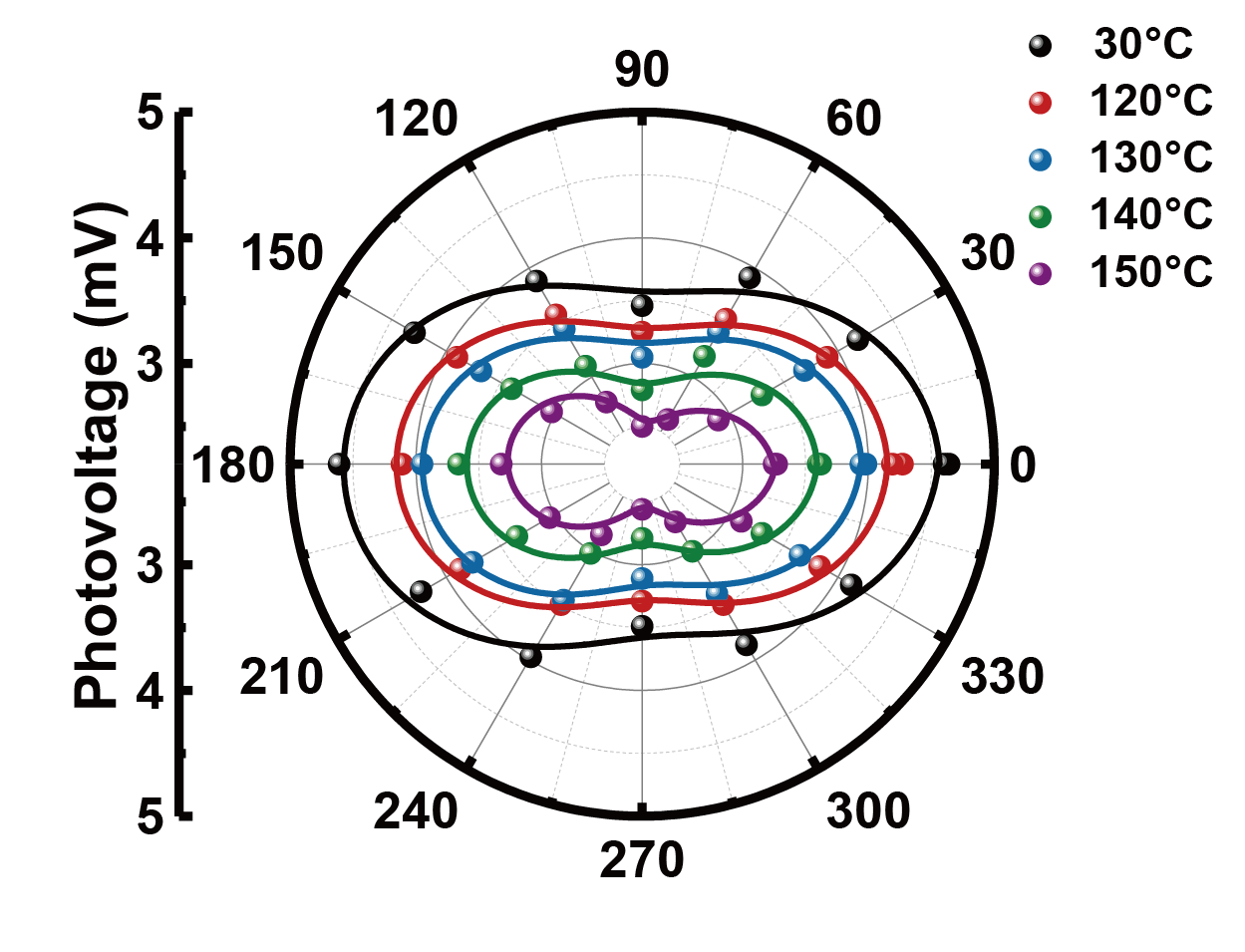


**Figure S22.** Polar plot of the photovoltage of I-shaped PDs at different temperature.


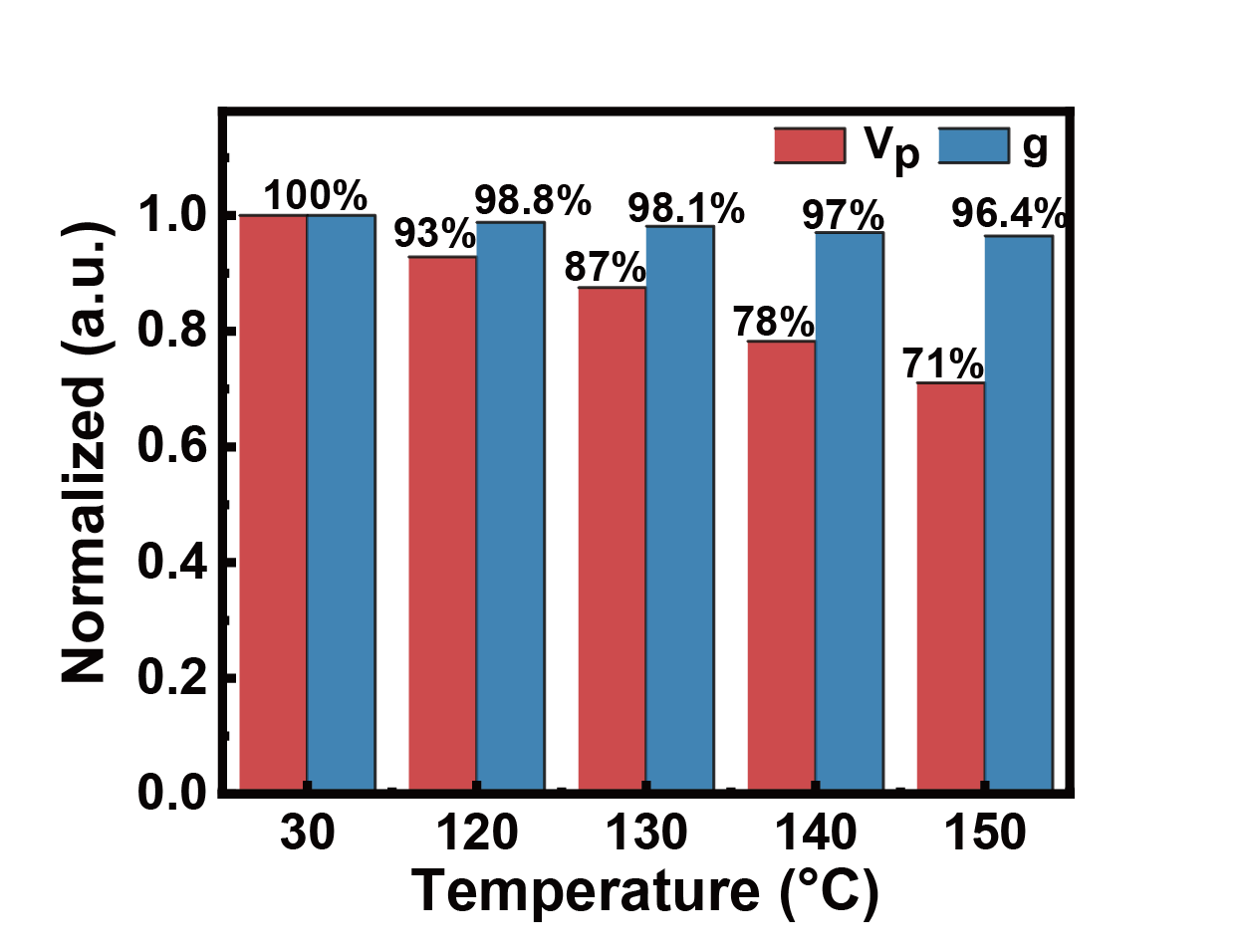


**Figure S23.** Photovoltage and polarization ratio of I-shape PDs at different temperature.


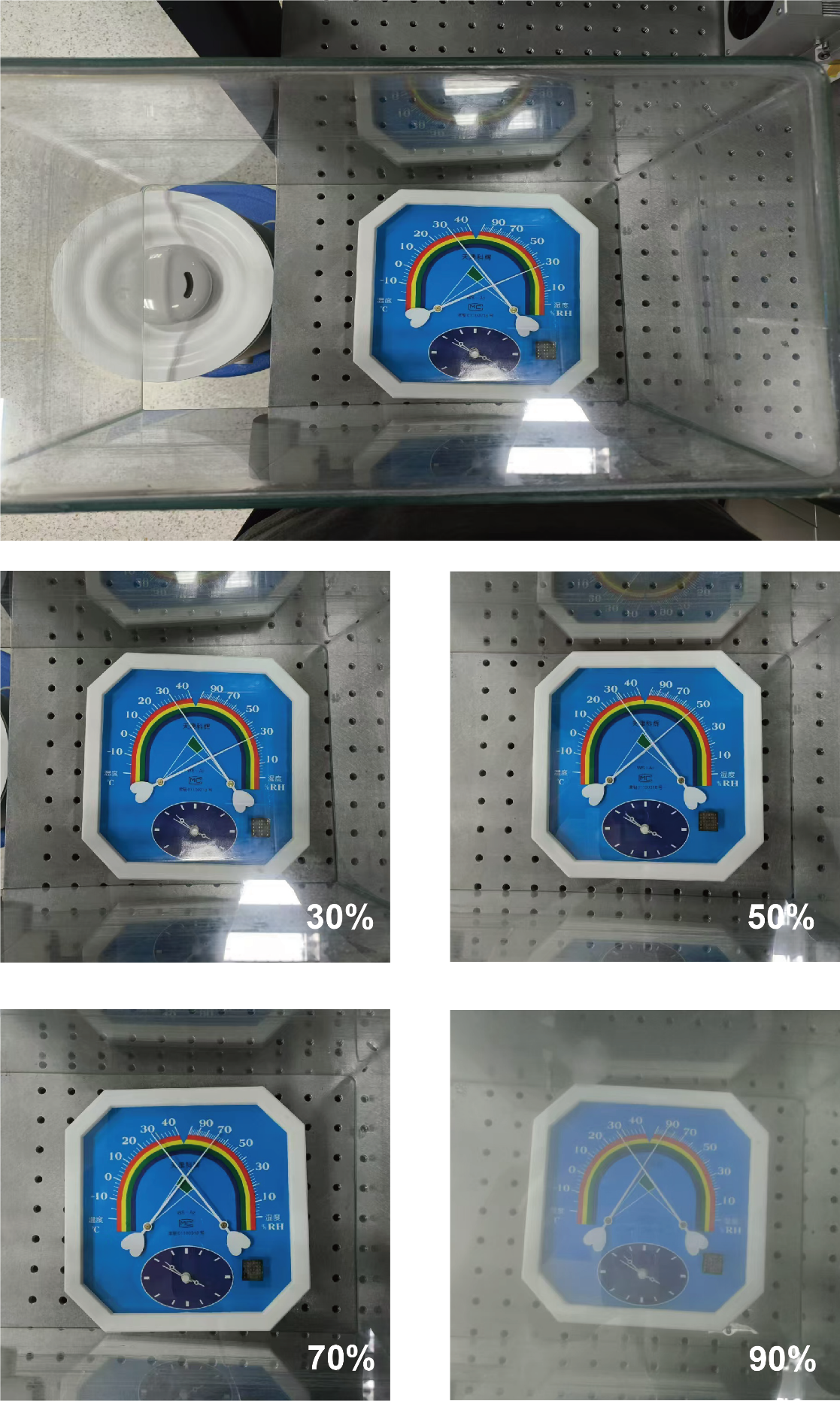


**Figure S24.** High humidity accelerated aging experiment (T-shaped).


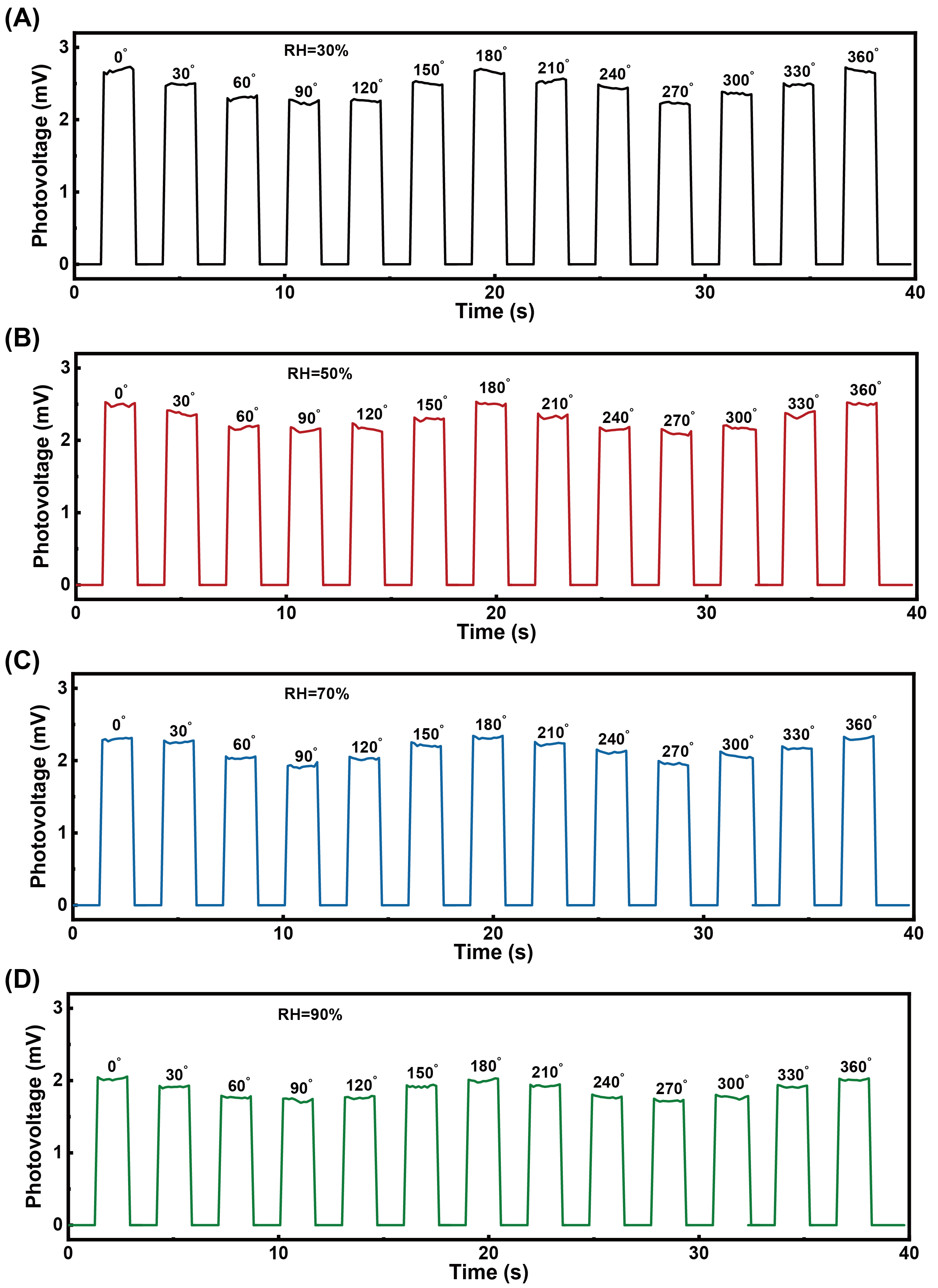


**Figure S25.** The photovoltage of T-shaped PDs measured after standing for one hour in an environment with humidity of 30%, 50%, 70% and 90%.


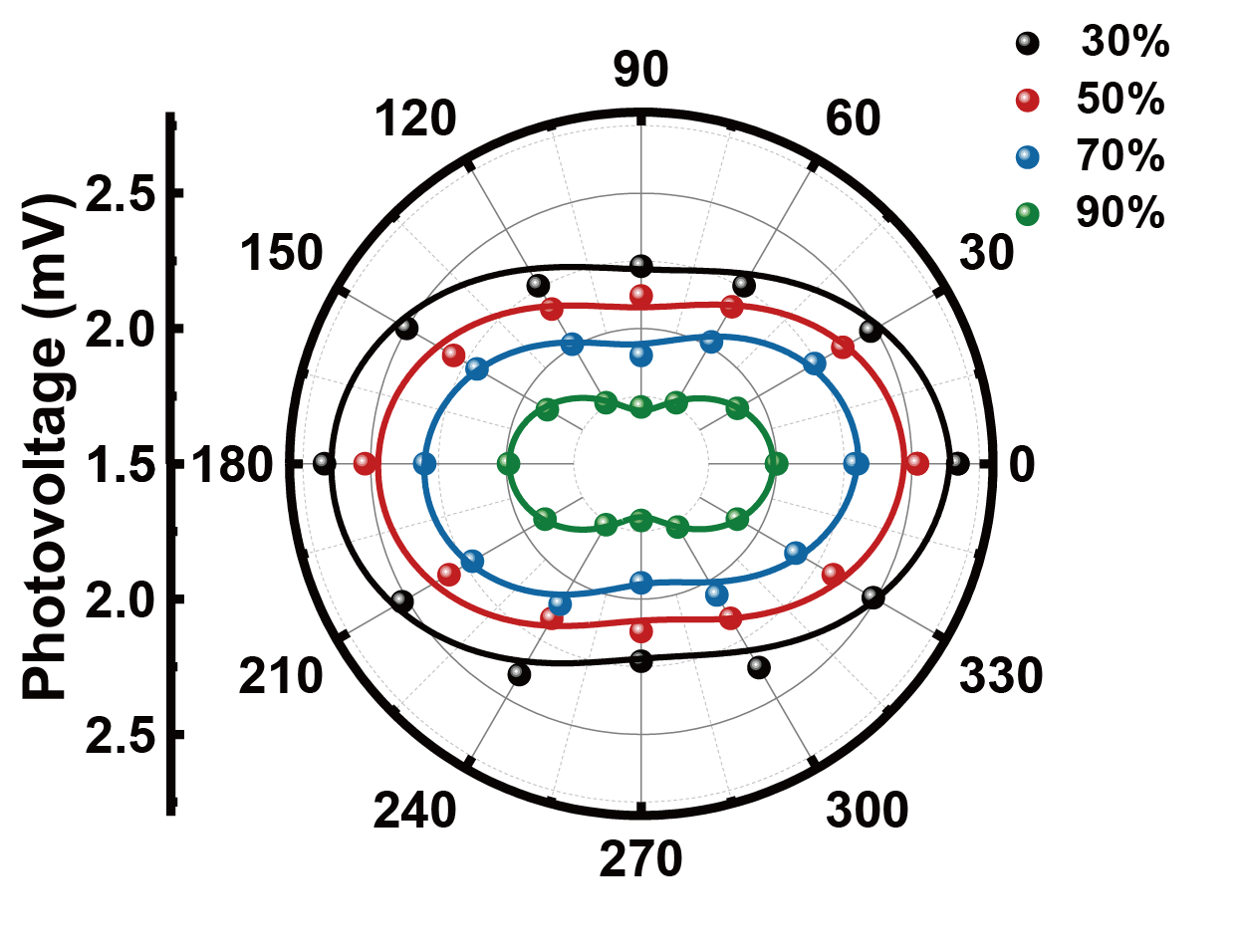


**Figure S26.** Polar plot of the photovoltage of T-shaped PDs at different humidity.


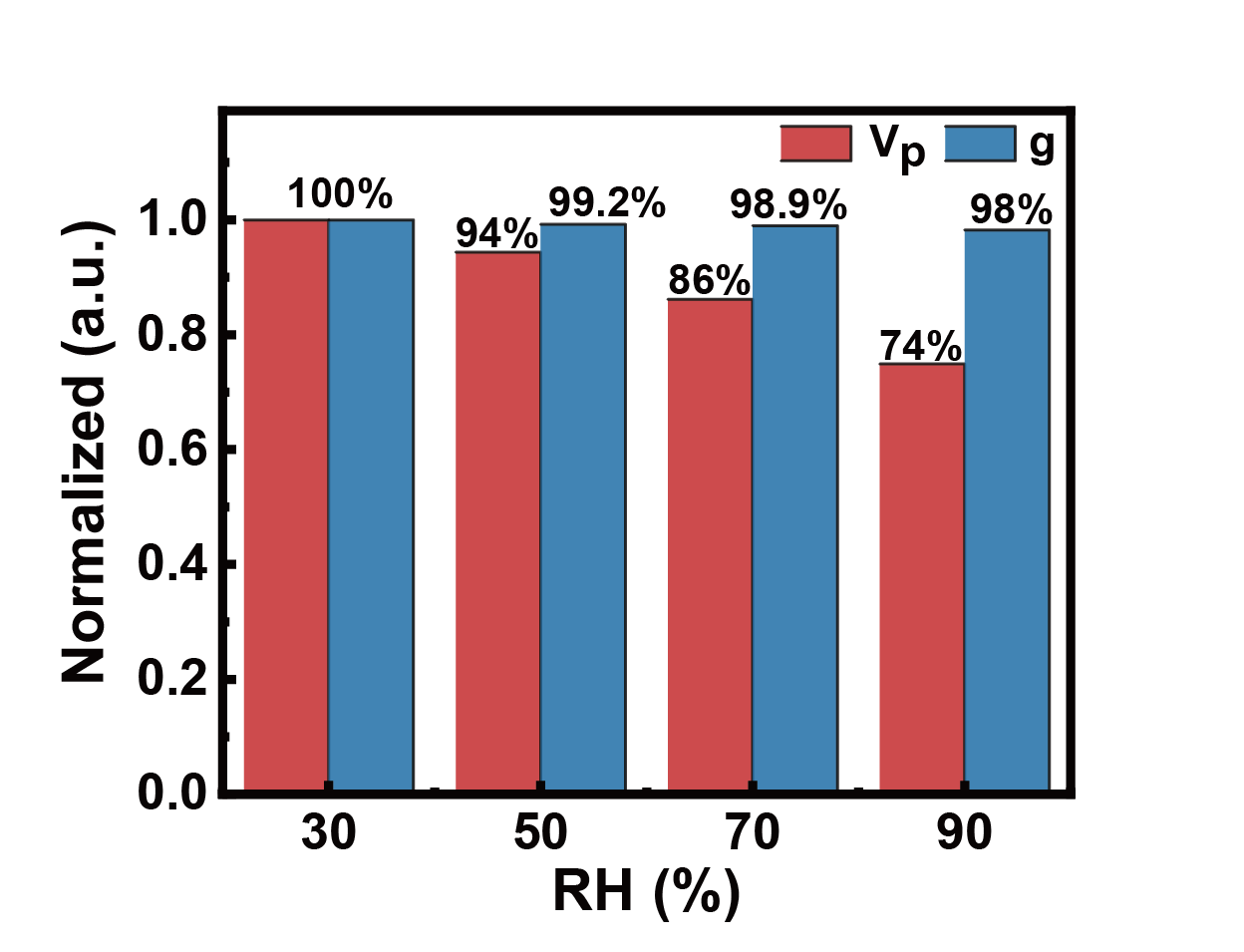


**Figure S27.** Photovoltage and polarization ratio of T-shape PDs at different humidity.


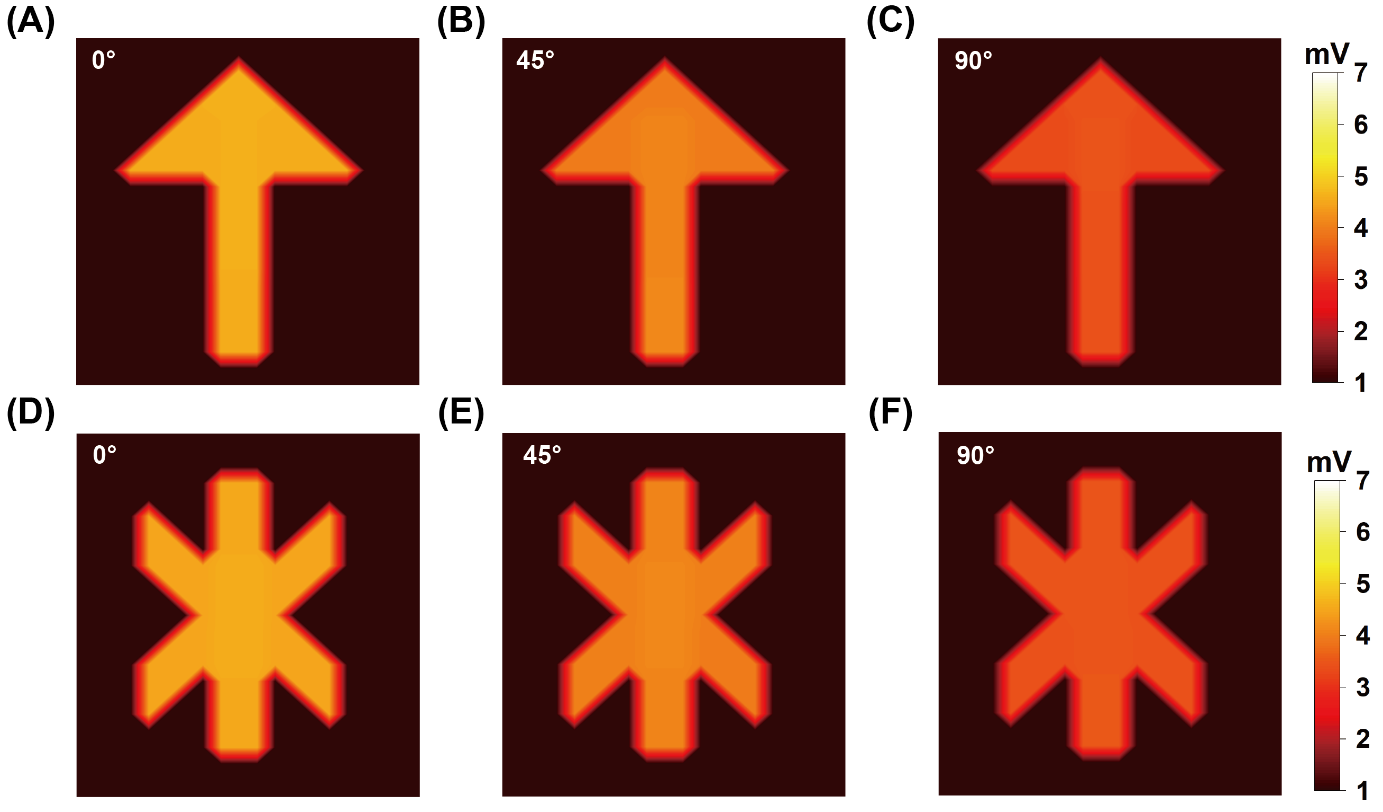


**Figure S28.** Imaging patterns of ‘↑’ and ‘*’ under 0.1THz light at 0 deg (left) 45deg (middle) and 90 deg (right).

**Table S1.** Performance of typical PDs.

| Description | *λ* | *R* | *NEP* (nW/Hz^1/2^) | *g* | Response time | Stability | Ref |
| --- | --- | --- | --- | --- | --- | --- | --- |
| BP | 0.29 THz | 1.7 V·W^−1^ | 0.1 | -- | -- | -- | 1 |
| AgNSF/CNTF | 2.54 THz | 0.0164 V·W^−1^ | 42.99 | -- | 6.6s | -- | 2 |
| Graphene/PtSe_2_ | 0.3 THz | 0.08 A·W^−1^ | 0.038 | -- | -- | -- | 3 |
| carbon nanotube | 2.54 THz | 11.7 mA·W^-1^ | -- | -- | 70 ms | -- | 4 |
| T_d_-MoTe_2_/mica | 2.52 THz | 0.53 mA·W^-1^ | 2.65 | 1.12 | 20 μs | -- | 5 |
| PdSe_2_ | 28 THz | 13 V·W^−1^ | 7 | 1.21 | 50 μs | -- | 6 |
| CsPbI_3_/CsPbBr_3_ | 650 nm | 0.125 A W^-1^ | -- | -- | 0.7 ms | 85%  @15d | 7 |
| (PEA)_2_PbBr_4_ /(PEA)_2_PbBr_4−x_ I_x_ | 365 nm | 13.5 AW^-1^ | -- | 5.6 | -- | 81%  @144d | 8 |
| Golay cells | 18~8000um | 1x10^5^V/W | 0.14 |  | 30ms |  | 9 |
| CsFAMA /Au metasurface | 0.1 THz | 94 V·W^−1^ | 5.03×10^-3^ | 1.38 | 138 μs | 96%  @240d | This work |

1. Wang M, Tian W, Cao F, et al. Flexible and self‐powered lateral photodetector based on inorganic perovskite CsPbI_3_-CsPbBr_3_ heterojunction nanowire array. Advanced Functional Materials. 2020; 30(16):1909771.

2. Viti L, Hu J, Coquillat D, et al. Efficient Terahertz detection in black-phosphorus nano-transistors with selective and controllable plasma-wave, bolometric and thermoelectric response. Scientific Reports. 2016; 6(1):20474.

3. Wang L, Han L, Guo W, et al. Hybrid Dirac semimetal-based photodetector with efficient low-energy photon harvesting. Light: Sci. Appl. 2022; 11(1):53.

4. Lv B, Liu Y, Wu W, et al. Local large temperature difference and ultra-wideband photothermoelectric response of the silver nanostructure film/carbon nanotube film heterostructure. Nature communications. 2022; 13(1):1835.

5. Liu Y, Yin J, Wang P, et al. High-performance, ultra-broadband, ultraviolet to terahertz photodetectors based on suspended carbon nanotube films. ACS applied materials interfaces. 2018; 10(42):36304-36311.

6. Yang Q, Wang X, He Z, et al. A Centimeter‐Scale Type‐II Weyl Semimetal for Flexible and Fast Ultra‐Broadband Photodetection from Ultraviolet to Sub‐Millimeter Wave Regime. Advanced Science. 2023; 10(17):2205609.

7. Dai M, Wang C, Ye M, et al. High-performance, polarization-sensitive, long-wave infrared photodetection via photothermoelectric effect with asymmetric van der Waals contacts. ACS nano. 2022; 16(1):295-30553.

8. Li S-X, Feng J-C, An Y, et al. Flexible, self-powered, and polarization-sensitive photodetector based on perovskite lateral heterojunction microwire arrays. Photonics Research. 2023; 11(12):2231-2241.

9. TYDEX, Golay, http://www.tydexoptics.com/products/thz_devices/golay_cell/ (accessed: September 2024).
